# What determines cell size?

**DOI:** 10.1186/1741-7007-10-101

**Published:** 2012-12-14

**Authors:** Wallace F Marshall, Kevin D Young, Matthew Swaffer, Elizabeth Wood, Paul Nurse, Akatsuki Kimura, Joseph Frankel, John Wallingford, Virginia Walbot, Xian Qu, Adrienne HK Roeder

**Affiliations:** 1 Department of Biochemistry and Biophysics, Center for Systems and Synthetic Biology, University of California, San Francisco, 600 16th St, San Francisco, CA 94158, USA; 2 Department of Microbiology and Immunology, University of Arkansas for Medical Sciences, Little Rock, AR 72205, USA; 3 Cell Cycle Lab, Cancer Research UK, London Research Institute, 44 Lincoln's Inn Fields, London, WC2A 3LY, UK; 4 Laboratory of Yeast Genetics and Biology, The Rockeller University, 1230 York Avenue, New York, NY 10065, USA; 5 The Francis Crick Institute, Euston Road 215, London, NW1 2BE, UK; 6 Cell Architecture Laboratory, Structural Biology Center, National Institute of Genetics, Yata 1111, Mishima, Shizuoka 411-8540, Japan; 7 Department of Biology, University of Iowa, 129 E. Jefferson Street, Iowa City, IA 52242, USA; 8 HHMI & Molecular Cell and Developmental Biology, University of Texas, Austin, 78712, USA; 9 Virginia WalbotDepartment of Biology, Stanford University, Stanford, CA 72205, USA; 10 Xian Qu, Cornell University, 244 Weill Hall, 526 Campus Rd, Ithaca, NY 14853, USA; 11 Cornell University, 239 Weill Hall, 526 Campus Rd, Ithaca, NY 14853, USA

## The big question of cell size

### Wallace F Marshall

For well over 100 years, cell biologists have been wondering what determines the size of cells. In modern times, we know all of the molecules that control the cell cycle and cell division, but we still do not understand how cell size is determined. To check whether modern cell biology has made any inroads on this age-old question, *BMC Biology *asked several heavyweights in the field to tell us how they think cell size is controlled, drawing on a range of different cell types. The essays in this collection address two related questions - why does cell size matter, and how do cells control it.

Why do cells care how big or small they are? One reason cell size matters is that the basic processes of cell physiology, such as flux across membranes, are by their nature dependent on cell size. As a result, changes in cell volume or surface area will have profound effects on metabolic flux, biosynthetic capacity, and nutrient exchange. A second reason is that the basic machinery of cell division in eukaryotes relies on microtubules, both to form the mitotic spindle and position it properly relative to the cortex. Because of the dynamic properties of microtubules, they are able to probe a limited range of lengths, and if cells get too big or too small, the mitotic apparatus may have difficulty working. Very small cells could not form a proper spindle, and very large cells could not coordinate their divisions during cleavage. This idea is elaborated in essays by Frankel and by Kimura, who discuss the apparent upper and lower limits on cell size with respect to cell division machinery. Finally, in both animals and plants, cells must fit together like puzzle pieces to form tissues and organs, and that means that a cell has to have a size appropriate to its position within the overall tissue, a topic discussed by Wallingford in the context of animal development.

Given that cell size is important, how can a cell control how big it is? In terms of 'design principles' for a size control system, the most fundamental question is whether cells need to know how big they are in order to regulate size. The simplest model is one in which cell mass grows at some rate determined by biosynthetic reactions (the rate could be dependent on cell size or not), and as they are growing, the cells divide at some constant frequency set by the cell cycle clock. Such a scheme would not require cells to ever actually know how big they are, but as discussed by Swaffer, Wood, and Nurse for yeast cells, experimental evidence rejects this simple model and suggests instead that cells can measure their own size and regulate the timing of cell division accordingly. This leads to the idea that cells can measure size, possibly by reading out intracellular gradients. But as discussed by Young and by Qu and Roeder, mechanical properties of the cell surface and of cytoskeletal elements can also play a role in determining size.

At the end of the day regulation of cell size may prove to be the combined result of several mechanisms operating in parallel, and that may be one reason it has been hard to study.

## Bacteria: appearances matter!

### Kevin D Young

The most obvious characteristic of bacteria is that they are small. Really small. As in requiring microscopes of high magnifying and resolving power to see them. So it surprises people to learn that the volume of these normally tiny cells can differ by as much as 10^6^- to 10^8^-fold, from the tiniest 0.2 μm cells of the *Pelagibacter *SAR11 clade that fills the oceans [1] to the monstrous genera *Thiomargarita *and *Epulopiscium *in which some species measure over 600 to 700 μm in length or diameter and are visible to the naked eye [2-4]. Of course, large bacteria are an extreme minority, with most known bacteria falling somewhere between 0.4 and 2 μm in diameter and 0.5 and 5 μm in length (though many grow as filaments that can be tens or hundreds of times this long). Another conceit is that bacteria are boring, at least in morphological terms. But this is just because most of us rarely encounter bacteria outside of what are usually brief episodes of disease, and the shapes of these common bacteria are admittedly pretty lame, being, as they are, no more than tiny cylinders. However, on a more global scale, bacterial shapes range from the plain (rods, spheres, strings) to the outlandish (branched, curved, coiled, spiraled, star-shaped), to the truly bizarre (fluted and tentacled) [5]. Given this range of possibilities, what determines the morphology of any one bacterium?

The first determinant is, as always, evolutionary. Bacteria cope with at least six fundamental selective forces that have some degree of control over the size that will best suit them to survive in particular environments. Specifically, bacteria adopt certain sizes and shapes so they can import nutrients most efficiently, meet requirements imposed by cell division, attach themselves to external surfaces, take advantage of passive dispersal mechanisms, move purposefully to pursue nutrients or avoid inhibitors, or avoid predation by other organisms [5,6]. Fundamental to all these considerations is that bacteria must accumulate nutrients that reach them by diffusion alone [7]. A basic tenet is that for such cells to exist the ratio of their surface area to cytoplasmic volume has to be quite high. Therefore, to maximize this ratio, most bacteria produce cells in the 0.2 to 10 μm size range and some organisms extrude long, exceedingly thin appendages to harvest nutrients present in low concentrations [8]. Because of this reliance on diffusion, those bacteria that reach near-millimeter size do so by employing clever morphological tricks. For example, some deploy their cytoplasm as a thin film around the outer rim of a large internal vacuole, creating a cell that looks very much like the skin of a balloon [2,9]. Others localize tens of thousands of chromosomes around the periphery of their cytoplasm, in near contact with the cell surface, so that each genomic equivalent 'governs' a volume approximately equal to that of a more normal, smaller cell [4]. Where a particular bacterium will eventually land in this size universe depends on other selective forces, which basically revolve around a bacterium's need to put itself in position to reach any nutrients at all versus the need to defend itself against becoming a nutrient for others.

The second determinant of bacterial morphology is mechanical, a factor that encompasses the biochemical mechanisms that do the heavy lifting of constructing cells of defined sizes and shapes. The current consensus is that morphology is determined primarily by molecular machines that synthesize the rigid cell wall. Three major types of machines are available. One, directed by the protein FtsZ, is responsible for nucleating the process of cell division and is shared by all bacteria, while the other, directed by the protein MreB and its homologues, is responsible for cell elongation in rod-shaped bacteria [10-13]. The third, first recognized by the activity of the CreS (crescentin) protein of *Caulobacter crescentus*, is responsible for creating the curved cells of this organism and the more regular shapes of other bacteria [14,15]. In a series of conceptual surprises, it was realized that FtsZ is a homologue, and perhaps progenitor, of the eukaryotic cytoskeletal protein tubulin [16,17], that MreB is a homologue of actin [18,19], and that CreS and its relatives are homologues of intermediate filaments, a third class of eukaryotic cytoskeleton proteins [14,15]. Though the structural similarities are clear, these proteins have been co-opted to perform different functions in bacteria. One last curiosity deserves mention: some classic metabolic enzymes also moonlight as cytoskeletal filaments that affect bacterial shape, a discovery with potentially far-reaching implications [20,21]. Finally, these basic tools can be modified, supplemented or differentially regulated to create morphologies from the simple to the quite complex.

There is room here to give only three brief examples of how rod-shaped bacteria control their overall size by varying cell length. The first involves *Escherichia coli*, a plain cylindrical rod that is normally about 1 μm in diameter and 2 μm long. In this organism, the future division site is determined by at least two mechanisms, each of which inhibits the polymerization or function of FtsZ and thus regulates when and where cell division occurs. First, driven by the MinD and MinE proteins, the MinC inhibitor oscillates back and forth between the two polar ends of the cell, taking approximately 1 to 2 minutes per cycle [22,23]. This behavior creates a time-averaged MinC concentration gradient that is highest at the poles and lowest near mid-cell. As the cell elongates, the concentration near the cell's center is reduced until it becomes so low that FtsZ can polymerize and initiate cell division. Therefore, cell size (as measured by length) is determined by the amount of MinC - larger amounts produce longer cells. Conceptually, this is eerily similar to the mechanism that regulates cell length in rod shaped fission yeast, as described by Swaffer *et al*. in this Forum article (below). Though there are biochemical differences, in this eukaryote cell length is regulated by a concentration gradient of Pom1 that is highest at the poles of a growing cell. Division is therefore inhibited until the cells become long enough so that the concentration of Pom1 at the cell center drops low enough to allow division.

The second way *E. coli *regulates cell length is by a 'nucleoid occlusion' mechanism [24]. Here, the SlmA protein binds to specific DNA sequences, and the SlmA-DNA complex prevents cell division by inhibiting FtsZ. Interestingly, SlmA binding sites are distributed around the chromosome except near the area where DNA replication terminates. During chromosomal segregation the two origins are pulled to either pole, and the two termination regions remain near the cell center, where they are the last to be replicated and separated. This means that as replication ends and when segregation is almost complete there will be a dearth of SlmA near mid-cell, at which time FtsZ will no longer be inhibited and can trigger division. Again, note how similar this is to the kind of mechanism that may explain how chromosomal ploidy determines cell length in yeast (see the contribution from Swafer *et al*. in this Forum article, below).

Recently a third, and surprising, mechanism was discovered by which cell length is tied to the metabolic status of the cell. *Bacillus subtilis*, a rod shaped bacterium about 1 to 2 μm in diameter and 5 to 10 μm in length, is longer when incubated in a nutrient-rich medium and shorter when nutrients are limited. Although it sounds simple, the question of how bacteria accomplish this has persisted for decades without resolution, until quite recently. The answer is that in a rich medium (that is, one containing glucose) *B. subtilis *accumulates a metabolite that induces an enzyme that, in turn, inhibits FtsZ (again!) and delays cell division. Thus, in a rich medium, the cells grow just a bit longer before they can initiate and complete division [25,26]. These examples suggest that the division apparatus is a common target for controlling cell length and size in bacteria, just as it may be in eukaryotic organisms.

In contrast to the regulation of length, the MreB-related pathways that control bacterial cell width remain highly enigmatic [11]. It is not just a question of setting a specified diameter in the first place, which is a fundamental and unanswered question, but maintaining that diameter so that the resulting rod-shaped cell is smooth and uniform along its entire length. For some years it was thought that MreB and its relatives polymerized to form a continuous helical filament just beneath the cytoplasmic membrane and that this cytoskeleton-like arrangement established and maintained cell diameter. However, these structures seem to have been figments generated by the low resolution of light microscopy. Instead, individual molecules (or at the most, short MreB oligomers) move along the inner surface of the cytoplasmic membrane, following independent, almost perfectly circular paths that are oriented perpendicular to the long axis of the cell [27-29]. How this behavior generates a specific and constant diameter is the subject of quite a bit of debate and experimentation. Of course, if this 'simple' matter of determining diameter is still up in the air, it comes as no surprise that the mechanisms for creating even more complicated morphologies are even less well understood.

In short, bacteria vary widely in size and shape, do so in response to the demands of the environment and predators, and create disparate morphologies by physical-biochemical mechanisms that promote access to a huge range of shapes. In this latter sense they are far from passive, manipulating their external architecture with a molecular precision that should awe any contemporary nanotechnologist. The techniques by which they accomplish these feats are just beginning to yield to experiment, and the principles underlying these abilities promise to provide valuable insights across a broad swath of fields, including basic biology, biochemistry, pathogenesis, cytoskeletal structure and materials fabrication, to name but a few.

## The puzzling influence of ploidy

### Matthew Swaffer, Elizabeth Wood, Paul Nurse

Cells of a particular type, whether making up a specific tissue or growing as single cells, often maintain a constant size. It is usually thought that this cell size maintenance is brought about by coordinating cell cycle progression with attainment of a critical size, which will result in cells having a limited size dispersion when they divide. Yeasts have been used to investigate the mechanisms by which cells measure their size and integrate this information into the cell cycle control. Here we will outline recent models developed from the yeast work and address a key but rather neglected issue, the correlation of cell size with ploidy.

First, to maintain a constant size, is it really necessary to invoke that passage through a particular cell cycle stage requires attainment of a critical cell size? If cells grow linearly - that is, the rate at which they accumulate mass in unit time is constant regardless of the mass of the cell - and if the time between successive cell divisions is maintained by a fixed timer, then cells will maintain size homeostasis. In successive generations all cells will slowly tend towards an average size [30]. However, work from both fission and budding yeast has shown this not to be the case [31]. Firstly, the variation in sizes at division of both yeast species is too small to be accounted for by such a process [31]. Secondly, cell cycle arrest of fission yeast results in enlarged cells that exhibit significantly shortened subsequent cycles [32]. This rapid reversion to the original cell size indicates the presence of a size correction mechanism. Similarly, in budding yeast, cells born smaller than normal spend longer in G1 until they reach a critical size [33]. There is also no evidence to suggest that yeast cells accumulate mass in a simple linear way for extended periods of time [34-37]. Therefore, there is a mechanism that monitors cell size and uses this information to regulate progression through events of the cell cycle. In the case of fission yeast this occurs primarily during G2 [38,39] but can operate in G1 [39,40], and for budding yeast it occurs during G1 [33,41].

**Figure 1:**
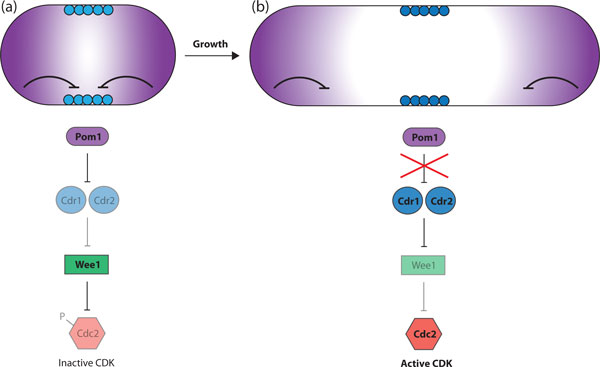
**The Pom1 gradient model for length sensing in fission yeast**. **(a) **An early G2 cell. Pom1 protein emanating from the cell tips (purple) inhibits Cdr1/Cdr2 in the cortical nodes (light blue circles). Wee1 is therefore active and carries out inhibitory phosphorylation of Cdc2. (**b) **A late G2 cell. Pom1 concentration at the medial site is decreased. At a critical threshold, Cdr1/Cdr2 are no longer inhibited (dark blue circles) and so the Wee1 inhibition of Cdc2 is lifted. The active CDK drives mitotic entry.

Two different molecular mechanisms for size control have been proposed for the two yeasts. In fission yeast, Cdc2 (Cdk1) kinase activity drives entry into mitosis and thus determines the length of G2 [42]. Wee1 catalyzes inhibitory phosphorylation of Cdc2 on Tyr15 and is antagonized by the phosphatase Cdc25 [42-46]. Cell size information is transduced via Cdr1 and Cdr2, inhibitors of Wee1 that localize to cortical nodes at the center of the cell [47]. Pom1 is a kinase that inhibits Cdr1/Cdr2, thus alleviating inhibition of Wee1 [47,48]. A gradient of Pom1 emanating from the cell tips inhibits G2/M until cells reach a critical length, and as cells are rod-shaped this is correlated with cell size. Pom1 is delivered to and associates with the plasma membrane at the cell ends. Pom1 autophosphorylation results in membrane dissociation, generating the Pom1 gradient, with a high concentration at the tips decreasing towards the cell center [49]. In a small early G2 cell there is sufficient Pom1 at the cortical nodes to inhibit Cdr1/Cdr2, preventing mitotic entry (Figure 1a). As cells grow and elongate, Pom1 concentration at the medial site becomes progressively lower, Wee1 is eventually inhibited by Cdr1/Cdr2, and cells commit to mitotic division [47,48] (Figure 1b). In this way the size of the cell regulates mitotic entry. However, this is unlikely to be the whole story because in cells where Cdc2 Tyr15 phosphorylation is prevented from occurring (thus bypassing Pom1-mediated regulation) cell size homeostasis is maintained, albeit with a broader size distribution [50]. This indicates other unknown mechanisms operate to measure size and integrate this information into cell cycle control.

In budding yeast, size control operates at 'start', a G1 event that commits the cell to cycle at a given size [33,41]. This is thought to operate by a protein synthesis-rate sizer mechanism involving the G1 cyclin Cln3 [31,51,52]. Cln3 is a dose-dependent activator of start [53-55], and is rapidly degraded [56]. Its high turnover rate means that the amount of Cln3 should be a direct reflection of the current rate of protein synthesis within the cell [57]. Since the number of ribosomes is indicative of cell size, protein synthesis rate will correlate with cell size. Therefore, only once a certain cell size is reached will there be sufficient Cln3 to drive the transition through start. This is thought to involve the activation of the transcription factor SBF by Cln3-CDK-mediated inhibition of Whi5, an SBF inhibitor [58,59]. A major problem with using protein synthesis rate as a proxy for cell size is that as the cell gets larger and more Cln3 is produced, the corresponding increase in cell volume should dilute out the protein, keeping it at a constant concentration. This appears to be the case for Cln3, as its relative abundance does not significantly change as cells grow during G1 [55]. To overcome this problem it has been proposed that Cln3 import into a nucleus of fixed size would allow the cell to 'measure' the absolute amount of Cln3 [51]. However, it has been shown for both budding and fission yeast that nuclear volume increases with the size of the cell [60,61], so this particular model remains incomplete and still requires a fixed 'standard' against which to measure Cln3.

Both of these models are interesting but, at least in their simplest form, they cannot account for a close to universal aspect of cell size: that is, the almost directly proportional increase in size at division that is observed as ploidy increases [62,63]. This relationship holds within a ploidy series of a single species, as well as across species [60,64]. This observation indicates that somehow cells can monitor their ploidy and integrate this information into the cell size-monitoring mechanisms. In general terms we envisage two types of model by which this might operate: either the cell makes a specific amount of a critical component according to ploidy, or it measures the amount of a given factor against ploidy.

An example of the former model would be if the transcript of the critical component were produced as a single pulse at a specific time in the cell cycle. The size of this burst of transcription would be a direct function of copy number, and as such a reflection of ploidy. If the gene product is stable and acts to inhibit division, as a cell grows, this fixed amount will be diluted down. Below a certain threshold concentration, its inhibitory effect is alleviated and division is permitted [65]. With an increase in ploidy there would be a requirement for a cell to be proportionately larger before the threshold is reached. If any gene were to operate in such a copy number-dependent inhibitory manner, it would be expected that a heterozygous diploid of such a gene would produce half as much protein (with no compensation) and therefore be the size of a haploid. It would be interesting to screen for genes behaving in this manner, but to our knowledge no such gene has yet been described. This may suggest that the model is an oversimplification, and such a mechanism might involve a number of interacting factors resulting in greater redundancy and adding robustness to the system.

An example of the second type of model would be a genomic titration mechanism [65,66]. This model invokes a protein that is maintained at a constant concentration, and which binds sites in the genome. As the cell grows larger, the absolute amount of this factor increases and thus more genomic sites become occupied. A critical threshold size is reached when a certain number of sites are bound. The occupancy of these sites could functionally drive a cell cycle transition - for example, by regulating transcription. Alternatively, this threshold could be a point of saturation at which no more sites are available to bind and the factor is free in the nucleoplasm or cytoplasm. The unbound factor could execute a pro-division function, which could even involve binding another DNA sequence for which it has lower affinity. A doubling in ploidy would be accompanied by a doubling in the number of DNA sequences for the protein to bind. This would impart the requirement on the cell to be twice the size before the occupancy threshold is surpassed. These are merely examples of the two types of models that could allow ploidy to regulate cell size and other variants are also possible [65].

Is it possible to modify the two proposed molecular mechanisms described above for yeast size control to take account of the effects of ploidy? With respect to the fission yeast Pom1 gradient model, perhaps the amount of a critical component in the network could be determined by gene copy number. In principle this could be the inhibitor, Pom1, although this is unlikely as deleting one copy of *pom1 *in a diploid does not reduce cell size to that of a haploid (Jacqueline Hayles, personal communication). Turning to the Cln3 activator model, it has recently been shown that Cln3 can bind to SBF binding sites across the genome [67]. Introduction of additional SBF binding sites increases cell size at 'start' in a Cln3-dependent manner [67]. It is plausible that this allows the cell to measure the amount of Cln3 against the genome, so only when a fixed number of sites are occupied is division permitted, as per the genome-titration model discussed above. This provides a possible solution to the aforementioned problems with the Cln3 protein synthesis rate sizing mechanism, as well as a means for ploidy to regulate size control directly. Other mechanisms, operating outside of the known size control network, that take account of ploidy could also be envisaged.

The universality of cell size scaling with ploidy means that ploidy should be taken account of when considering cell size-sensing mechanisms. It may also imply that there is conservation of the mechanisms involved, although whether this conservation exists at the level of molecules or of control network architecture remains to be seen.

## Physical limits of cell size for embryonic cell division in *Caenorhabditis elegans*

### Akatsuki Kimura

Early embryos are a good model for studying the relationship between cell size and intracellular organization. Blastomeres can exhibit various sizes during embryonic cell division since cells divide without cell growth during this phase. In addition, embryonic cells are generally large, which makes these cells useful models for microscopic observation. Thus, transparent *Caenorhabditis elegans *embryos represent an ideal model for investigating these relationships [68-71]. Here, I argue, based on prior studies in *C. elegans *and other systems, that cell size may be limited by the physical properties of the cell. In order to proliferate, the cell has to divide, and for faithful cell division, molecular machinery, such as the mitotic spindle, must be constructed at the right position and with the correct size. This may not be accomplished in extremely large or small cells due to the physical properties of macromolecules, such as microtubules and chromosomes.

**Figure 2:**
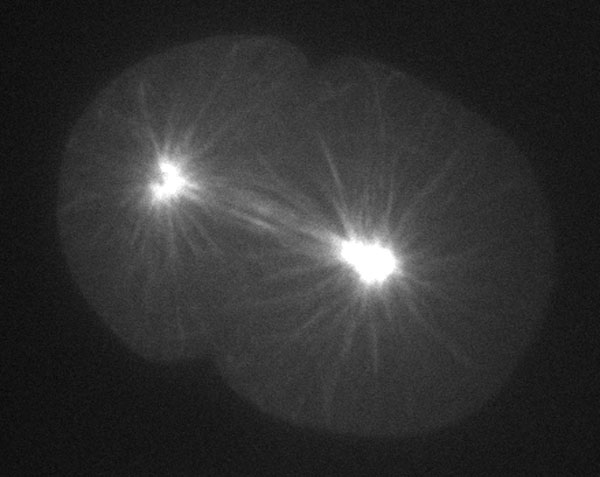
**Microtubules in *C. elegans***. An image of microtubules in an embryonic cell - astral microtubules from the spindle reach the cell cortex.

Positioning of the mitotic spindle at the cell center is critical for symmetric cell division, as it defines the origin of sister chromatid segregation and the position of the cell division plane [72]. The centrosome is a major organizing center of the microtubule cytoskeleton and in animal cells often includes the poles of the mitotic spindle. Centrosomes have the ability to position themselves at the cell center [73], enabling the mitotic spindle also to position at the cell center [74,75] (Figure 2). This central positioning of the centrosome is accomplished through the function of the microtubule cytoskeleton [76,77]. Several mechanisms have been proposed to describe how microtubules bring centrosomes to the cell center, including pushing of the cell cortex and pulling by motor proteins [74,77-88]. The mechanisms mediating centrosome centration may differ among species, especially among species with different cell sizes [83]. Recent studies have supported the idea that the cytoplasmic pulling force is a major driving force for centrosome centration in animal cells [75,78,81,86,89]. Importantly, for all proposed mechanisms, microtubule-dependent centration of the centrosome must be facilitated by microtubules, which grow from the centrosome and span throughout the cytoplasm to find the geometrical center of the region [87,88].

**Figure 3:**
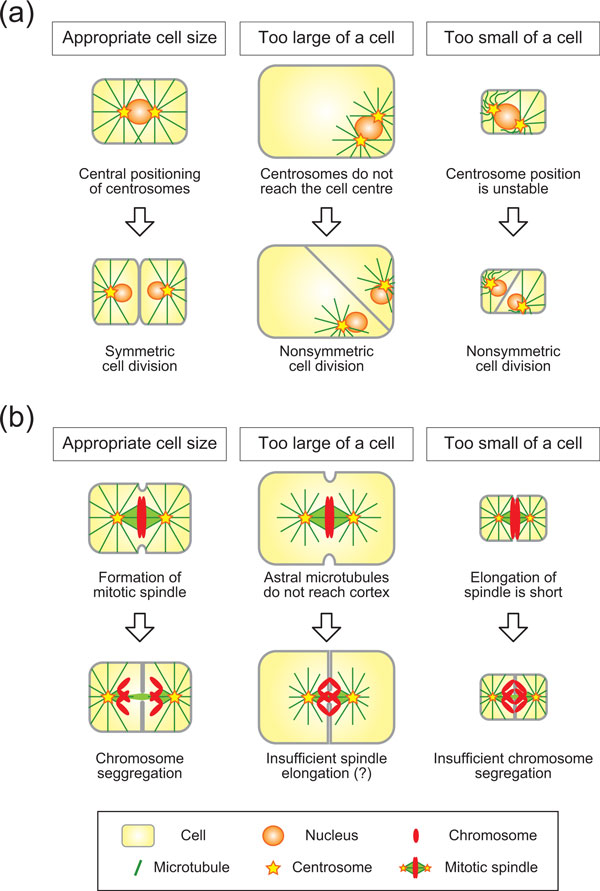
**Possible scenarios in which centrosome centering (a) and spindle elongation (b) set the upper and lower limit of cell size**. **(a) **If the cell exceeds the upper limit of size, the centrosome, and consequently the mitotic spindle, cannot position at the cell center, leading to nonsymmetrical cell division (middle panels versus left panels). If the cell falls below the lower limit, the centrosome may not stably position at the cell center due to the excess elastic forces of the microtubules (right panels versus left panels). **(b) **If the cell exceeds the upper limit, astral microtubules do not reach the cell cortex, potentially leading to insufficient spindle elongation. If the cell falls below the lower limit, there may not be sufficient space for accurate chromosome segregation compared to the size of the cell's chromosomes.

If the cell is too large compared to the length of microtubules, the centrosome will not position at the cell center (Figure 3a, middle panels). Since the microtubules grow and shrink in a stochastic manner known as dynamic instability [90], the mean length of microtubules (*n_av_*) is defined by the velocities of growth (*v_+_*) and shrinking (*v_-_*), the frequency of switching from growth to shrinking (*f_+-_*) and vice versa (*f_-+_*), as *n_av _*≈ (*v_-_v_+_*)/(*v_-_f_+- _*- *v_+_f_-+_*) [91]. According to this equation, mean lengths are estimated to be in the micron range based on experimentally measured dynamic instability parameters *in vitro *[92] and *in vivo *[93], which is consistent with observed *in vivo *microtubule lengths [91,94]. Interestingly, this length is comparable to the size of ordinary animal cells, suggesting that the length scale of a microtubule is related to the size of the cell. Therefore, the length of microtubules may define the upper limit of cell size. This idea has been supported by experimental shortening of microtubule length. When cells were treated with microtubule depolymerizing drugs or partial knockdown of microtubule polymerizing molecules, centrosomes did not reach the cell center and the cell division plane was positioned in an asymmetric manner [76,95,96]. Interestingly, studies have demonstrated that centrosomes can find the cell center in extremely large cells, such as newly fertilized frog embryos [97] (see the contribution from Dr Frankel in this Forum article, below). In large embryos, a large microtubule aster that expands throughout the cell is formed and centers the centrosome toward the cell center, possibly due to cytoplasmic pulling forces [89]. In an *in vitro *centering experiment using microfabricated chambers, it was demonstrated that efficient elongation of microtubules to reach the boundaries of the chamber was critical for robust positioning of the microtubule aster at the center of the chamber [88]. These studies collectively support the idea that cell radius cannot exceed the maximum length of microtubules for cell proliferation.

How are the lower limits of cell size defined? The centrosome may not correctly center if the cell is too small compared to the length of the microtubules. Because of the elastic properties of microtubules, short microtubules generate strong pushing forces when the growing tips encounter obstacles such as the cell cortex. Due to the reactions of these pushing forces, the centrosome will be subjected to strong forces from multiple microtubules, which may destabilize the positioning of the centrosome (Figure 3a, right panels). This idea is based on an elegant *in vitro *centering experiment using microfabricated chambers combined with theoretical analyses [79,98,99]. This experiment was performed in a cell-size chamber (a square with about 20 μm on a side), but the length of the microtubules were long due to the lack of shrinking; many microtubules reached the chamber boundaries, exerting pushing forces against the chamber walls. Under these conditions, microtubule asters moved away from the center of the chamber [79] or failed to reposition to the cell center after relocation [99]. This off-center movement was restored by promoting microtubule shrinkage [99]. Shortening of the average length of the microtubule by promoting shrinkage resulted in fewer microtubules reaching the cortex, thereby generating less pushing force, and stably positioned the aster at the geometrical center [99]. This experiment indicated that, in small cells where many short (and thus rigid) microtubules reach the cell cortex, the centrosome cannot stably position at the cell center.

In addition to the physical properties of microtubules such as the length and stiffness, other properties may also define the limits of cell size. Elongation of the mitotic spindle during anaphase is known to depend on cell size; the larger the cell, the longer and faster the spindle elongates. To date, this trend has only been demonstrated in the *C. elegans *embryo [68,69]; however, it may represent a general trend in other cells. If a spindle elongates only for a short distance in small cells, the separation of sister chromatids may not be enough to segregate them completely. In the yeast *Saccharomyces cerevisiae*, artificially elongated chromosomes become more condensed at anaphase to ensure complete segregation of the chromatids [100]. This observation indirectly implies that the spindle must elongate to a certain distance in order to segregate chromosomes with a certain size [101]. Within a given genome size, there should be a lower limit to the size of condensed chromosomes, which may further define the lower limit of the elongation of the mitotic spindle, thus defining the size of the cell (Figure 3b, right panels).

Elongation of the mitotic spindle may define the upper limit of cell size as well. Since elongation is partly driven by pulling astral microtubules from the cell cortex [102,103], if astral microtubules do not reach the cell cortex in large cells, the elongation of the spindle is impaired, potentially causing insufficient chromosome segregation (Figure 3b, middle panels). To my knowledge, there has been no experimental evidence to support this idea so far. However, when the cortical pulling force was impaired in *C. elegans *embryos, we were able to shorten spindle elongation, albeit not completely [69]. Chromosomes appear to manage segregation under these conditions. The identification of genes responsible for the remaining elongation may allow us to test whether impairing the interaction between astral microtubules and the cell cortex leads to chromosome segregation defects.

In this text, I discussed a basic and simplified view of the relationship between cell size and the material properties of macromolecules comprising the mitotic machinery. As the biology always has to deal with diversity, an individual cell may have its own unique mechanism to set cell size and to accomplish cell division. Nevertheless, the diversity in cell size among species is far smaller than that in body size, suggesting common constraints for the majority of cell types. I believe understanding such common constraints will reveal the basic design principle of cell architecture.

## The largest dividing cells: are they alike?

### Joseph Frankel

*'Physical extremes, in this case a very large cytoplasm, are always interesting in biology' *[97]

#### How can we frame a useful inquiry about the upper limits of cell size?

If one asks which cell is the largest, one comes up with a list of highly diverse candidates. Those on the centimeter scale include the ostrich egg measured at 8 cm by EB Wilson [104], the 3 to 5 cm unicellular stalked marine alga *Acetabularia *[105], and various giant shelled (testate) amoeboid denizens of the deep-sea bottom, including the 3 cm 'living fossil' *Gromia sphaerica *[106]. The multinucleate green alga *Caulerpa *is, however, the champion unicellular organism, with a tubular stolon extending to a length of one meter or more, as well as a remarkable degree of internal differentiation and a propensity for rapid vegetative growth [107].

The modes of propagation of *Acetabularia *and *Gromia *are typical of most marine giant single-celled organisms. They do not appear to undergo binary fission, but instead become multinucleate for at least part of their life cycle, after which they produce large numbers of flagellated gametes [108,109], each of which includes only a small portion of the overall cytoplasmic mass of the large parent cell [105,108]. The same is true for *Caulerpa *during its episodes of sexual reproduction [110]. Thus, these giant cells can endow their progeny with DNA and elementary organelles such as mitochondria and (in algae such as *Acetabularia *and *Caulerpa*) also chloroplasts [109,110], but they almost certainly do not transmit any significant portion of their cytoplasmic organization to their individual offspring.

All of these enormous cells have some device for escaping the consequences of their large size when they reproduce, either by cleaving only a small portion of their mass, as in reptilian or bird eggs, or by subdividing all or part of their large cell bodies to produce swarms of diminutive progeny, as in centimeter- and meter-scale marine unicellular organisms such as *Acetabularia *or *Caulerpa*. When producing reproductive cells, such organisms probably do not need to make any global assessment of their overall dimensions and organization. In my view, the interesting upper size limit is the largest size at which a cell can carry out such a global assessment and then make use of this assessment to perpetuate itself by dividing into two daughter cells similar in form and structure to itself. We can then ask what this size limit actually is.

An interesting 'test case' is provided by two amoebae, *Amoeba proteus *and *Chaos chaos*, that are known to be very closely related [111]. *Amoeba proteus *is roughly 500 μM in length when actively moving [112], is uninucleate, and goes through a fairly typical process of mitosis and cytokinesis [113]. By contrast, the giant amoeba, *Chaos chaos *(A.K.A. *Chaos carolinensis, Pelomyxa carolinensis) *measures 1 to 5 mm when extended [112] and is multinucleate. While these nuclei undergo synchronous mitoses [114], the subsequent cell division is very atypical: it simultaneously produces between two and six daughters, and the several hundred nuclei of the parent cell are segregated apparently at random among these division products [115]. This process, called 'plasmotomy' by Kudo [114], is a far cry from the typical mitotic cell division found in the closely related but much smaller uninucleate *Amoeba proteus*.

This example introduces a problem: large cells typically need more DNA than normal-sized cells to support their metabolic and synthetic needs. In extreme cases, they may become multinucleate, in which case the multinucleate condition itself may generate an obstacle to normal cell division. Even more profoundly, the extensive endoreduplication of DNA that is often found in large differentiating cells in plants is closely associated with cessation of cell division [116] (also see the contribution from Qu and Roeder in this Forum article).

Two distinct types of large cells have found different ways of circumventing these obstacles to binary fission. The ciliates do so by possessing a single large polygenomic macronucleus that maintains the vegetative functions of the cell and divides amitotically together with the cell in which it resides. Cleaving eggs do not need multiple nuclei or polytene chromosomes because they possess abundant stored maternal mRNA and (generally) delay the onset of zygotic nuclear transcription, thereby allowing several initial rounds of nuclear division and cytoplasmic cleavage to occur in rapid succession.

I shall here consider these two examples of the consequences of progressive enlargement, in ciliates and in amphibian eggs, the first analyzed using a microsurgical approach and the second following a comparative approach. Comparison of these two cell types indicates that there is a fairly constant upper size limit to whatever organization permits normal cell division, but that the specific organization appears to be markedly different in these two types of cells.

#### A microsurgical approach: grafting *Stentor*

**Figure 4:**
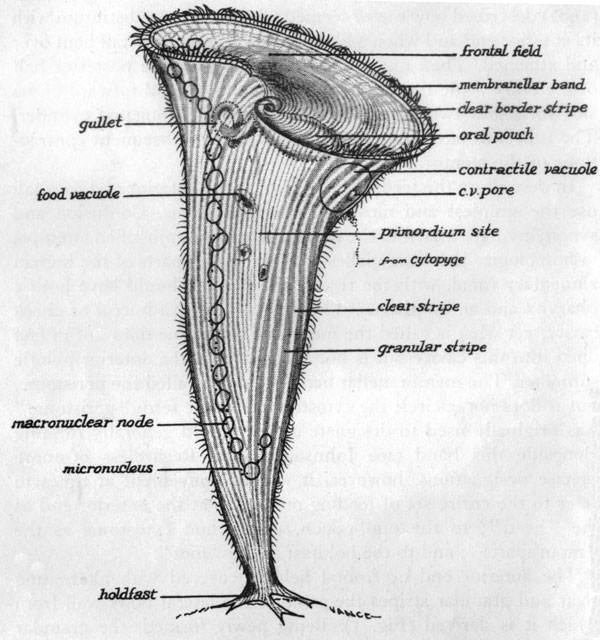
**A descriptive diagram of *Stentor coeruleus***. All of the features shown are on the cell surface, except for the macronuclear nodes, the micronuclei, and the contractile vacuole, all three of which are located just beneath the surface. The cortical fibrillar system, not shown in the diagram, is located within the clear stripes between the granular (pigmented) stripes. From Figure 1 of [118]. Image courtesy of Biodiversity Heritage Library. http://www.biodiversitylibrary.org

The paradigm for the microsurgical approach has been provided by the largest ciliate, *Stentor coeruleus*, measured by Morgan [117] at 1.4 to 2.8 mm in length when fully extended (Figure 4). Vance Tartar carried out a systematic program of intra-species grafting to discover the limits of its recovery of normal form and of its capacity for cell division. Comprehension of Tartar's findings requires awareness of three basic facts about *Stentor*: first, 'that cell shape is an expression of the cortical stripe pattern' ([118], p. 211), which in turn is closely associated with the pattern of ciliary rows that are interdigitated among the stripes; second, that *Stentor *is capable of maintaining one, two or three (but not four or more) parallel sets of major cortical landmarks (oral structures and oral primordia) without severely compromising its normal cell form; and third, that the consistency of its internal cytoplasm makes it possible for an experimenter, with some skill and practice, to fuse whole stentors together in any orientation, or to graft parts of one stentor onto another stentor [118].

The overall result of Tartar's analysis of 272 combinations of whole stentors grafted together in random orientations is best summarized in Tartar's own words: 'Fusion masses of two to four stentors were generally capable of recovering fully or approximately the monaxial, normal body shape and of dividing thereafter...to give single, doublet, and triplet progeny. In the larger grafts (involving random fusion of more than four whole stentors) both shape recovery and cell-division were lacking. ...the masses were unable to even begin fission; only artificial cutting up of the masses into approximately normal volumes produced normal singles' ([119], p. 564). These fusion masses (which could not feed) 'lived for about the same period of time as starved controls' ([119], p. 569) implying that their failure was most likely due to starvation resulting from their inability to feed rather than to anoxia resulting from their increased mass and reduced surface-to-volume ratio. This inability to feed was a consequence of the inability of these large masses of artificially fused stentors to form normal oral structures, for reasons to be explored below.

**Figure 5:**
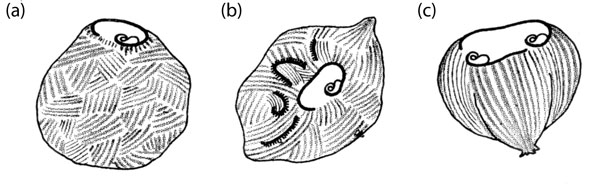
**Random slicing into the cell cortex ('minceration') facilitates the integration of a large fusion mass**. **(a) **A graft of six whole stentors fused together, minced, plus an implanted oral apparatus. **(b) **After two days, four small oral primordia were formed at irregular sites. **(c) **By the sixth day, the fusion mass reconstituted a doublet with normal oral structures and a cell shape resembling that of a normal single cell. From Figure 12 of [120].

Further analysis revealed that surgical manipulation of the cortical pattern itself could influence the recovery of normal form. Microsurgical disarrangement of large blocks of cortex resulting from grafts of large portions of stentors in unnatural arrangements often brought about bizarre structural outcomes, whereas stentors demonstrated 'an astonishing capability...to regenerate and to reconstitute the normal, orderly arrangement of the ectoplasmic pattern...after all of the complex ciliary, contractile, conductive and other differentiations of the ectoplasm have been cut into tiny pieces scattered at random' ([118], p. 224). Further, such thorough disorganization ('minceration') of the cortex of *Stentor *increased the upper size limit for attainment of normal form: a minced six-mass (a group of six stentors artificially fused together followed by random slicing into the cell cortex of the fusion mass using a fine glass needle) 'had succeeded, as un-minced six-masses do not, in reconstituting a doublet with a single-cell shape' ([120], p. 200) (Figure 5). Even a 25-mass (that is, 25 stentors artificially grafted together), after minceration, could manage a partial recovery of normal form, although with no oral differentiation (see below).

These observations appear paradoxical until one realizes that the graft-fusions were, as Tartar pointed out, mostly random. Thus, an un-minced fusion complex had large blocks of normally juxtaposed cortex rearranged in a coarse crazy-quilt disorder. The minceration effectively made the pieces of the quilt much smaller. These small pieces then could rotate on the fluid endoplasm, and later come into alignment with other such pieces, with selection probably favoring homopolar alignments, thereby enabling a gradual reconstruction of a coherent and fairly normal cortical pattern [120].

But then what is the basis of the upper size limit? Here we need to introduce one further element of *Stentor *lore. That is the notion of gradients. The German investigator Gotram Uhlig, who carried out his own experimental analysis of *Stentor *morphogenesis independently of Vance Tartar in the 1950s, explained most of his results on the basis of two interacting gradients, one basal-apical and the other circumferential [121]. Tartar subsequently adopted Uhlig's basal-apical gradient to account for certain otherwise inexplicable results of one of his own experiments [122]. The relevance of this postulated gradient in the current context is that one of its principal expressions is the induction of mouthparts at the posterior end of the oral apparatus of *Stentor *as it develops within the primordium site shown in Figure 4.

Returning to the *Stentor*-masses resulting from the fusion of whole stentors in random orientations, minced six-masses were able to form normal oral apparatuses with mouthparts (oral pouch and gullet, Figure 4), whereas minced 25-masses were not; they instead produced 'two garlands of adoral cilia without cytostomes' (that is, membranellar bands lacking the oral pouch and gullet) superimposed on a rough approximation of the normal *Stentor *form ([119], p. 559). Failure to form proper mouthparts within such 'garlands' was typical for the large fusion-masses, which Tartar attributed to 'the presence of numerous cell axes running in random directions and canceling each other in their polar influences' ([118], p. 215). But failure would also be expected if the sheer size of the large *Stentor-*masses rendered them unable to reconstitute a normal basal-apical morphogenetic gradient. Recalling that if the large masses were cut into smaller pieces they could then regenerate normal single stentors, one may ask whether the insuperable dilemma faced by these large masses is due to their excessive structural complexity or to their large size or to some combination of the two. This issue may be ripe for re-investigation with modern means for visualizing cytoskeletal organization.

Vance Tartar did not supply scale-bars for his published drawings of operated stentors. While he acknowledged his failure to obtain 'super-giant normal stentors' ([119], p. 567), the normal-appearing doublet that emerged from a fused and minced six-mass (Figure 5) must nonetheless have been at least transiently larger than a normal *Stentor*. Assuming an extended length of a normal *Stentor *in the order of 2 mm, I would then estimate a maximum *linear *(basal-to-apical) dimension for form regulation in *Stentor coeruleus *at somewhere close to 3 mm.

#### A comparative analysis: early cleavages in amphibians that lay extra-large eggs

In animals, the largest dividing cells are eggs. The largest totally cleaving (holoblastic) eggs are found among amphibians. The most familiar frogs (such as *Rana pipiens *and *Xenopus laevis*) and salamanders (such as *Ambystoma mexicanum *and *Triturus*) lay eggs with a diameter between 1 and just over 2 mm [123,124]. However, other species found in both of the two major amphibian orders (anurans and urodeles) produce eggs that range up to 10 mm in diameter. This large size of the egg is associated with direct development and/or parental care [125,126]. Unlike the situation with Tartar's grafted stentors, we know that even the largest eggs somehow manage to complete their development. Nonetheless, we can still ask whether their early cleavages remain regular and orderly as egg diameter increases. The simple answer is that they do not.

The pattern of cleavage has recently been investigated in two large-egged amphibian species: one marsupial frog and one land-dwelling salamander. Early cleavages in the marsupial frog *Gastrotheca riobambae*, which has an egg diameter of 3 mm, are holoblastic, extremely slow, commonly asynchronous, and frequently asymmetric in that they do not cut the egg into equal halves. Hence, when different eggs at the same cleavage stage are viewed from above the animal pole, each one has a different pattern of blastomeres, and the number of complete blastomeres at any given time is commonly not in the series of 2^n ^[127]. The plethodontid salamander *Ensatina eschscholtzii*, the largest appropriately studied amphibian egg, has a 6 mm diameter, and has taken a further step in the direction of meroblastic (partial) cleavage. Early cleavages (beyond the first two) are extremely irregular, and 'cleavage initially occurs only in the animal pole, with no cleavage furrow visible in the vegetal pole until about the 16-cell stage' ([124], p. 3). This implies that progression of cleavage furrows from the animal into the vegetal region is either very slow or delayed, probably due to the high concentration of yolk in the vegetal hemisphere.

Collazo and Keller [124] discuss the modifications of cleavage in large-egg amphibians in the light of a distinction proposed by SJ Gould, between 'historical' and 'formal' developmental constraints. Historical constraints are ones that depend upon contingencies of ancestry and descent. Formal constraints exist independently of ancestry and are dictated by physical principles or restrictive structural relationships [128]. Collazo and Keller attribute most of the modifications of early development found in large-egged salamanders to historical constraints, because these are diverse in different lineages. However, they make an exception for the effect of egg size on early cleavage patterns, which they attribute to a formal constraint in the Gouldian sense. In their words, 'The fact that the asymmetries and asynchronies in early cleavage seen in these four species (the three others are from early 20^th ^Century descriptions of large-egged salamanders) are qualitatively similar and that these species represent two disparate salamander families suggests that large egg size and not phylogenetic relationship accounts for the differences in development from amphibians with smaller eggs' ([124], p. 8).

To a first approximation, the threshold in linear dimension between regularity and irregularity of amphibian cleavage appears to be somewhere between 2 and 3 millimeters - similar to the size threshold in capacity of *Stentor*-masses to regulate to the normal *Stentor *form (and concomitantly the normal capacity to divide). This might be a general size-limit in the capacity for a well-organized binary cell division.

#### Divergent organization: outside-in versus inside-out

Even if we accept that a similar upper size threshold exists for normal cell division in *Stentor *and in amphibian (and fish) eggs, it could still be that the similarity in these size thresholds is coincidental. This is especially likely because the respective roles of the cortex and the endoplasm in the cell's accommodation to large cell size appear to be opposite in the two types of cells.

In *Stentor *as in other ciliates, the cortical layer is structurally the most highly organized part of the cell. That is where the granular pigment stripes and the intervening cortical fibrillar system, including basal bodies, cilia, and accessory microtubular bands, are located. The nodes of the macronucleus adhere to the cortical layer and several small micronuclei are nearby [118] (Figure 4). An ultrastructural study of a closely related ciliate *(Blepharisma) *has shown that the mitotic division of the micronucleus is closed, with no trace of centrioles, centrosomes or astral fibers [129]. Tartar was able to remove 'practically all the endoplasm [of *Stentor*] by vigorous pipetting', after which the eviscerated stentors could 'regenerate and fill out the cell shape within a day' ([118], p. 108). Cell division in *Stentor *is a process that, to a large extent, is driven by the longitudinal growth and transverse segmentation of the cortical pattern [130].

**Figure 6:**
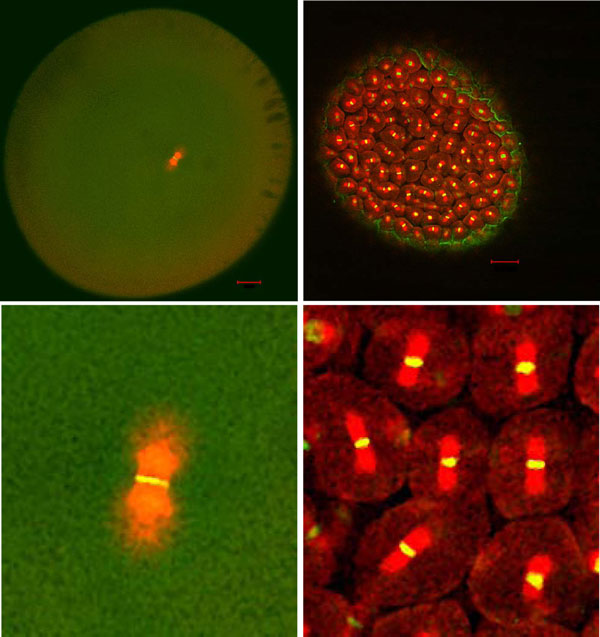
**Accommodations must be made**. The one cell *Xenopus *embryo (left) is over 1 mm across, while cells within the embryo several hours later (right) are closer to 50 microns. Mitotic spindles are shown in red and chromosomes in yellow. Complex mechanisms have evolved to allow for cellular functions such as cytokinesis and mitosis to be effective in cells of diverse sizes. Images are courtesy of Martin Wühr (Harvard).

The division of a fertilized frog egg could hardly be more different. Frog eggs are roughly spherical and have no obvious cortical differentiations, apart from the pigmented cap on the animal hemisphere [131]. The division apparatus is entirely internal. The mitotic spindle is small relative to the large size of the egg and becomes located deep within the cell (Figure 6, left). The asters, on the other hand, are dynamic structures that re-form at telophase of each of the early divisions, expand tremendously, and while expanding become centered by dynein-mediated pulling forces that act on the astral microtubules even before these microtubules touch the cell surface. Cell division furrows then ingress 'where the interaction zones between telophase asters touch the cortex' ([89], p. 2043). Thus, while the cortex is involved passively in the determination of the location of the fission zone (and actively in its subsequent ingression), the earlier dynamic processes, including the sensing of cell volume, are all endoplasmic. These mechanisms, based on the balancing of pulling forces from multiple locations in the cytoplasm rather than from the cortex, were proposed as adaptations that would allow asters to function properly in large-sized amphibian eggs [97], yet they also appear to function in the smaller eggs of the sea urchin and of the nematode worm *Caenorhabditis elegans *[75,81,86] (also see the contribution from Dr Kimura in this Forum article).

These studies were carried out on the egg of *Xenopus laevis*, which at a diameter of 1.2 millimeters is large as a cell and even as an egg, yet smaller than the eggs of many other frog species [126]. Therefore, one wonders whether the irregularities of cleavage that emerge as amphibian eggs get larger are in some way related to size limitations in the effective functioning of the centrosomal-centering and cleavage-site determining mechanisms that operate so efficiently in the *Xenopus *egg. This problem is discussed by Kimura in this Forum, and is illustrated in Figure 3. The postulated 'nonsymmetric cell division' resulting from the failure of centrosomes to reach the cell center (Figure 3, top center) is reminiscent of the irregular cleavage patterns observed in the very large eggs of the frog *Gastrotheca riobambae *[127] and the gigantic eggs of the salamander *Ensatina eschscholzii *[124].

In view of these major differences, we can also wonder whether the size limits of the two largest cleaving cell types, the large ciliate *Stentor coeruleus *and the even larger amphibian eggs such as those of *Ensatina eschscholtzii*, have anything at all in common. I think that they just might. In cleaving eggs, there are good reasons to believe that size limits are based on properties of microtubules (see above, as well as the contribution from Dr Kimura in this Forum article). While a consideration of microtubular systems of ciliates is beyond the scope of this contribution, these systems are known to be abundant and in part dynamic over the cell cycle, yet have been little investigated in the largest ciliates such as Stentor. It is just barely possible that there might be some underlying limit to the spatial extent over which microtubule-based cytoskeletal systems can organize and then reorganize themselves in the absence of internal cell boundaries. Further structural and molecular investigations of large minced *Stentor *grafts and the largest amphibian eggs might yield some interesting and unexpected insights into these and perhaps other unanticipated questions.

## Size matters, but in animals so does shape

### John Wallingford

I am nearly two meters tall, so the neurons linking my toes to my spinal cord are quite enormous. These neurons are well over twice the size of those belonging to my four year old daughter, even though her skin fibroblasts are probably about the same size as mine. This anecdote, unscientific though it may be, serves to illustrate two key facts. First, that in addition to the many problems of cell size control faced by unicellular organisms, animals face the added challenge of establishing and maintaining cell type-specific cell sizes. And second, that the need to control cell size over developmental time presents an additional hurdle.

The control of cell size in animals is of course a wide-ranging topic, and it is no surprise that key players in cell size control in unicellular organisms are also key players in animal cell size control. Genetic studies in *Drosophila *have revealed the key role of cell cycle regulators in controlling cell size, and the phosphoinositide 3-kinase pathways are also widely studied for their link to cell size in mammals [132,133]. However, given the deep conservation of such mechanisms, it is perhaps more interesting in this forum to discuss some less well-known, cell type-specific problems that arise at the interface of cell size control and development. In this respect, a consideration of developing amphibians provides some illuminating vignettes.

Some of the pioneering studies for the link between cell size and cell proliferation in animals were performed in salamanders, where Fankhauser noted that the increase in cell size in heteroploid animals was compensated for by decreases in cell numbers. Thus, he found that the salamanders and their constituent organs were all roughly the same size, be they diploid, triploid or even pentaploid [134]. Such compensatory effects are widespread in animals, as reflected by the more recent genetic studies in *Drosophila*, for example [132].

Like most animals, amphibians develop externally and without ongoing maternal nutrition, and so their eggs are packed with yolk. The enormous size of the one-cell frog embryo (>1 mm across [135]) is therefore a crucial facet of its lifestyle, but it also presents a problem. During cell division, these large cells must deploy specialized mechanisms for generating the enormous amounts of new plasma membrane to build the >500 micron-long nascent cleavage furrow [136]. Likewise, the mechanisms of mitosis have been modified to achieve proper chromosome separation in such a gigantic cell [89,97], even though the size of their spindles remains surprisingly small, capped apparently by an upper physical limit [137] (Figure 6). During these early stages, cell division is uncoupled from cell size [138], but these modifications - however crucial to the early embryo - are quickly abandoned. By the 12^th ^division, cells are a far more reasonable approximately 50 microns in diameter and links between cell size and cell division are put in place [138].

At this same time, another developmental landmark serves to illustrate the importance of cell size: after 12 divisions, *Xenopus *embryos engage the zygotic transcriptional machinery for the first time [139]. As in many other animals, this onset is determined by a nuclear-to-cytoplasmic volume ratio [140]. This ratio must necessarily be influenced not only by cell size, but also by nuclear size, so it is noteworthy that nuclear size, like organelle size generally, is not a simple reflection of overall cell size. Rather, recent *in vivo *studies in frog embryos combined with *in vitro *studies exploiting embryo extracts have identified factors in the cytosol that are crucial to the control of nuclear size [141]. These cytosolic factors are even more crucial than is ploidy [141], a result that in many ways parallels findings in yeast [60].

Similar experiments suggest that mitotic spindle length is also dependent on cytosolic factors [142]. In the smaller cells of later stage embryos, spindle length scales with cell size [137], and this scaling requires input from the actin cytoskeleton [143]. In larger cells, spindle length does not scale with cell size, and even in cytoplasm extracts *in vitro*, where spindles cannot be constrained by any physical cue, there are spindle-intrinsic cues that set a strict upper limit on length [137,144]. Collectively, these results not only illustrate some of the recent advances in our understanding of organelle and cell size in embryos, but they also highlight a general gap in our understanding at the intersection of developmental and cell biology: we have a fairly detailed picture now of many fundamental cell biological processes, but much of this picture is drawn from studies of relatively few cell types, many of which exist only in culture. Though comparatively sparse, *in vivo *studies consistently show that these fundamental processes vary from cell type to cell type in animals, but the factors controlling such cell type-specific modifications remain for the most part poorly defined.

**Figure 7:**
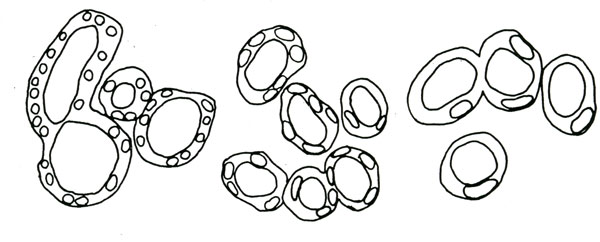
**Accommodations must be made again**. Animals have evolved mechanisms that maintain tissue size in the face of changing ploidy. These images from Fankhauser illustrate this point. Kidney tubules of the same size are constructed by larger and larger cells as ploidy increases, and cells must change their shapes so that the tubule diameter can remain constant. In the haploid animal, many nuclei can be observed around the tubule circumference, and so these cells have a columnar morphology. In the pentaploid animals, only one or two nuclei can be observed, and these much larger cells are flattened and squamous in order to enclose the same tubule diameter.

Finally, there is one issue of cell size control that may be unique to animals, and that is the impact of cell size on cell movement. Large-scale movements of individual cells are central to animal morphogenesis, and the last decade has seen huge leaps forward in our understanding of the molecular control of force generation during animal morphogenesis [145,146], but we know very little about how cell size influences these processes. This fundamental question was articulated by Fankhauser himself, who noted that radical changes in cell shapes were needed in order to generate normally shaped organ structures out of the much larger cells in heteroploid animals. One example he gave was the developing kidney tubule, where five or six cells of roughly columnar shape spanned the circumference in normal diploid animals. Only two of the larger cells in a pentaploid animal enclosed the tubule, and these cells were flattened and curved such that tubule diameter remained similar to that in diploids (Figure 7). This finding suggests that regulatory mechanisms are in place to sense cell size and adjust cell morphology accordingly in order to maintain tissue structure [134]. Conversely, mechanisms also exist to allow larger cells to form a larger but morphologically normal kidney, a situation called compensatory renal hypertrophy [147]. Such compensation commonly occurs in one kidney when the other is somehow compromised.

Recent molecular studies also provide clues to the interaction between cell size control and morphogenesis. For example, live imaging suggests that kidney tubule diameter is controlled in part by cell rearrangements, and when these are disrupted, tubules become dilated and cystic [148,149]. Similar cystic phenotypes have been linked to the Hippo pathway, a key signaling mechanism governing cell division and organ size control [150,151]. Likewise, the same genetic pathways that govern cell size control in normal development also govern renal hypertrophy [152,153]. So here again, detailed cell biological studies performed *in vivo *will be central to our efforts to understand the tangled interactions between cell size and morphogenesis in animals.

## Thinking inside the wooden box - classic views of cell size control in plants

### Virginia Walbot

Historically, botanists quantified various cellular shape and volume parameters and discovered a very tight correlation between nuclear DNA content (the C value), nuclear volume, and cell volume in meristematic cells with small vacuoles [154]. Over a diverse range of C values, including exemplar monocots with giant genomes, mid-range genome size monocots and dicots, and *Arabidopsis thaliana *with a tiny genome, the log cell volume is linearly related to log nuclear volume with a correlation of 0.99. There are numerous 'ploidy' series within species (or very close relatives) in nature and among horticultural derivatives. *Ficus *spp. trees, ubiquitous decorations in hotel and airport lobbies, are diploid, tetraploid, or octoploid. These ploidy levels are readily distinguished by comparing the size of the epidermal guard cell pairs or other epidermal cells. From a few centimeters distant, however, the trees are indistinguishable in architecture and leaf size (V Walbot, personal observation) despite the obvious fact that octoploid leaves contain fewer, larger cells.

In the Introduction to this Forum, Wallace F Marshall opines that cell physiology depends on cell size. This is true, perhaps, in cuboidal animal cells in which relative surface area declines with volume and the cytoplasm is served by a 'smaller' surface area. Because plant cells have vacuoles, however, gigantic diploid cells with very large vacuoles will have a tremendous surface area, and such cells will actually experience a much higher surface area per unit cytoplasm than a smaller cuboidal cell. In a ploidy series or in an organ with cells of various ploidy levels, the higher ploidy cells can have an even more favorable surface to cytoplasmic volume relationship.

Observations on living plants indicate that growth and most morphology do not depend on the number of cells or their size within an organ. A very striking observation concerns lethally irradiated seeds: upon germination the pre-existing cells of the embryo enlarge, generating a seedling that is remarkably normal despite the lack of cell division [155-157]. Not so surprising for the first leaf, which contained almost the full complement of cells, but each successive leaf starts with fewer cells yet achieves near normal morphology despite ridiculously large cells and abnormal anatomy. In a separate study, the same researchers applied colchicine to inhibit cell division in roots, yet single-celled lateral root primordia initiated in the normal location and grew in a manner paralleling the normal developmental pattern of multicellular lateral roots [158].

Yet, plant development is highly regular, and leaves and roots of similar size with approximately equivalent numbers of cells are produced by a population of seedlings. This regularity of developmental pattern is particularly high in reproductive organs such as the anther, in which stereotyped cell division patterns establish the tissues, and there is a high degree of similarity in cell numbers and volumes in different anthers [159]. For example, anther developmental outcome in maize is extremely similar in different inbred lines, although developmental mechanism may differ: in the inbred W23 the secondary parietal layer makes a physically symmetric periclinal division to establish the middle layer and tapetal cell layer while in inbred A619 this division is highly asymmetric. At the conclusion of cell patterning and growth, however, the differentiated middle layer and tapetal cells are virtually identical in these two inbred lines. This regularity of development is the basis for genetic screens to identify mutants with perturbations in cell enlargement or cell division. Despite the large number of mutants available and many measurements of growth parameters, the relationship between cell division, cell expansion, and their balance to establish cell size remains mysterious. Moving up to the scale of a tissue or organ, the cellular composition (number of cells and their sizes) can vary widely, depending on ploidy or treatments that modulate cell division and expansion.

Emergent patterns during plant growth is a topic that fascinated Alan Turing. This brilliant mathematician pondered the Fibonacci series intrinsic to spiral botanical patterns (pinecones, petals, and so on), although unfortunately nearly all of his botanical insights remained as unpublished manuscripts and notebooks. Thus, it is gratifying that today in addition to cytological and genetic study of plant development, mathematical modeling is enjoying a renaissance in plant biology, and the fundamental problem of growth control is one attractive target. Improvements in cell imaging - principally the use of confocal imaging and the use of *in vivo *fluorescent cell type markers - has generated copious data on cell numbers, sizes, and changes in dimensions over developmental time. These new datasets have sparked a renaissance in the application of mathematical modeling to plant growth. The integration of observation, computation, and simulation represents a new frontier in growth analysis, with cells at the heart of all three ways of thinking about tissue growth. In the next section, our current state of knowledge, tool kit, and challenges for these three paths are integrated and critiqued.

## Plant cell size control: all things considered

### Xian Qu, Adrienne HK Roeder

Plant biologists have long wondered about the mysterious phenomenon called 'compensation': when the cell number is decreased as the result of a mutation, the cell size increases, leading to the production of organs with nearly normal area [160]. This phenomenon raises two basic questions for plant biologists: how is the size of a plant organ controlled and how is the size of plant cells controlled? Although these interrelated puzzles have been extensively studied for many years, neither is fully understood. Two processes contribute to size control during organogenesis: cell division and cell growth. Cell size growth in plants is driven by increase in mass (reflecting macromolecular synthesis) and increase in volume (primarily through expansion of the cell wall and the central vacuole). The relationship between cell division, cell size, and organ size remains controversial, as illustrated by the contradictory hypotheses put forward in two recent computational models [161,162]. Here we will examine the contributions of cell cycle regulation, ploidy, macromolecular synthesis of cytoplasm, cell wall expansion, and developmental regulation in the control of plant cell size, focusing on examples from *Arabidopsis*.

Changing cell cycle regulator activity can alter the length of time that cells spend in growth phases G1 and G2 before dividing, thus affecting cell size. In *Arabidopsis *leaves and cultured cell lines, overexpression of *CYCLIN D3;1 *(*CYCD3;1*) triggers a quick transition into the mitotic cycle, reducing the proportion of cells in the G1 phase of the cell cycle, and decreasing cell size [163,164]. In contrast, slowing division with a dominant negative form of the *CYCLIN DEPENDENT KINASE A;1 *(*CDKA;1*) increases final cell size [165]. Likewise, expressing the CDKA;1 inhibitor *KIP RELATED PROTEIN1 *(*KRP1*) under the control of an epidermis-specific promoter results in slower epidermal division and somewhat increased epidermal cell size [162,166]. In contrast, overexpressing either *APC10 *or *CDC27*, which are two subunits of the anaphase-promoting complex/cyclosome (*APC/C*), increases cell division rates without decreasing the final cell size [167,168]. Thus, cell size can be altered by some cell cycle regulators, but is unaffected by others.

In many organisms, including plants, a strong correlation exists between ploidy, nuclear size, and the volume of the cytoplasm [169-171]. In young plant cells with small vacuoles, the cytoplasm fills the cell, so that ploidy correlates with cell volume. In contrast, in mature plant cells, the large central vacuole fills the volume, and the cytoplasm is restricted to a thin layer on the surface of the cell, such that the ploidy correlates with the surface area [172].

Polyploid cells are found outside of polyploid plants; diploid plants commonly contain polyploid cells produced through endoreduplication - a variant of the cell cycle in which cells stop dividing and instead continue to grow and replicate their DNA [171]. Endoreduplication causes an increase in both cell size and ploidy [172] and is often associated with specialized cell types. Endocycles and mitotic cell cycles can occur simultaneously during development in neighboring cells [162] and endoreduplication is a mechanism for cell enlargement. Many alterations inhibiting the cell cycle regulatory machinery cause increased endoreduplication in organs. Although the exact trigger that causes an individual cell to endoreduplicate is unknown, one speculation is that the cell needs to exceed a prior cell size checkpoint. One finding supporting this hypothesis is that overexpression of either of two D-type cyclins, *CYCD2;1 *or *CYCD3;1*, induces smaller cell sizes and blocks endoreduplication [163,173]. Once the putative cell size checkpoint is passed, endoreduplication can be switched on by various mechanisms. One such mechanism is reduction of CDKA;1/CYCLIN B activity to a level that fails to initiate mitosis but is still able to drive replication of DNA. For example, mitotic CDKA;1 activity can be reduced by elevated activity of cyclin-dependent kinase inhibitors of the KRP and SIAMESE families [174,175]. The SIAMESE protein has CYCLIN and CDKA;1 binding sites, and the protein can inhibit mitosis and stimulate endoreduplication in the *Arabidopsis *hair cells (trichomes) [176,177]. A second trigger of endoreduplication is ectopic DNA replication. *CDC6 *catalyzes assembly of the ORIGIN OF REPLICATION (ORC) complex, enabling DNA replication and overexpression of *CDC6 *increases endoreduplication in *Arabidopsis *[178].

In plants, the ratio of the ploidy to the cytoplasm remains constant, suggesting that cytoplasm can influence cell size as well. Cytoplasm increases through macromolecular synthesis. For example, *Arabidopsis *EBP1 is related to human ErbB-3, an epidermal growth factor receptor binding protein that enhances translation. Overexpression of the *EBP1 *gene in *Arabidopsis *increases cell growth [179], implying that increased protein synthesis correlates with increased cell size. Similarly, inhibiting protein degradation by mutation of the 26S proteasome subunit *REGULATORY PARTICLE AAA-ATPASE 2a *(*RPT2a*) [180] produces larger cells in *Arabidopsis *leaves [181,182], showing that optimizing proteasome activity levels is important for cell size control. As in many other organisms, macromolecular synthesis is regulated in *Arabidopsis *by the TARGET OF RAPAMYCIN (TOR) pathway [183]. The pathway regulates cell growth and metabolism in response to growth factors, nutrients, energy, and environmental conditions in yeast and mammals [184,185]. The *Arabidopsis *genome encodes only one *TOR *gene, and it regulates cell size, translation initiation, and ribosomal RNA synthesis [183,186].

Cell wall expansion is a major mechanism controlling plant cell size. The plant cell wall greatly confines the enlargement of plant cells because it consists of a rigid mesh of complex polysaccharides and a few structural proteins [187]. The cell wall grows by repeated stretching and polymer reconnection (termed stress relaxation): turgor pressure constantly exerts a stretching force on the cell wall, and the force is alleviated when polysaccharide polymers rearrange their interconnections to take on the new shape [188]. To maintain cell wall integrity, new polymers are synthesized simultaneously and added to the growing wall. Thus, proteins that modify the interconnections between polysaccharide polymers regulate plant cell growth. The *EXPANSIN *(*EXP*) family is one class of plant proteins that are thought to loosen cell walls by weakening the binding of polysaccharide polymers to one another [189]. In fact, adding active EXPANSIN proteins to dead cell walls is sufficient to cause their rapid extension [190]. Overexpression of *EXP10 *in *Arabidopsis *under the control of its own promoter results in larger leaves containing larger cells [191]. The extensibility of the cell wall is not uniform in all directions, and is limited by the reinforcement of the cellulose microfibrils embedded in the wall. The primary direction of expansion, and ultimately the shape of the cell, depend on the orientation and alignment of the cellulose microfibrils, which are controlled by the cytoskeleton just inside the plasma membrane of the cell. In vascular plants, a cellulose microfibril is synthesized by a cellulose synthase complex of integral plasma membrane proteins [187]. The cellulose synthase complex tracks through the plasma membrane along the cortical microtubules in such a way that microtubules determine the orientation of the new cellulose microfibrils in the cell wall, and thus control the direction of structural reinforcement [192]. In *Arabidopsis*, both microtubule- and microfilament-associated proteins facilitate normal cell expansion in different organs [193-195]. The cell wall presents a second challenge to plant cell growth in that the walls of neighboring cells are tightly connected. Because plant cells generally do not slip relative to one another, the growth of neighbors is coordinated along their adjoining walls. Nonetheless, heterogeneity of cell growth is not only still possible but common in individual cells, because portions of the cell wall in contact with different neighbors can grow with different rates [196].

The relationship between organ growth through turgor-induced cell wall expansion and organ growth through cytoplasm biosynthesis and cell division is unclear. As mentioned above, plant cells have a tremendous ability to increase their cell wall expansion to compensate for decreased cell division, resulting in an organ with normal size but extremely large cells. However, compensation fails when too few cells are available. Perhaps this was best illustrated by experiments in which cell division was completely blocked in wheat seedlings by gamma irradiation of the grains. Remarkably, the leaf primordia of these gamma plantlets still grow and produce first foliage leaves with the correct shape [155,197]. However, after ten days, the gamma irradiated plant leaves are only about 15% as long as unirradiated plants, despite greatly increased cell expansion [197]. These experiments indicate that there is a limit to cell wall extensibility in living plants and some cell division is required for organs to reach their normal size.

**Figure 8:**
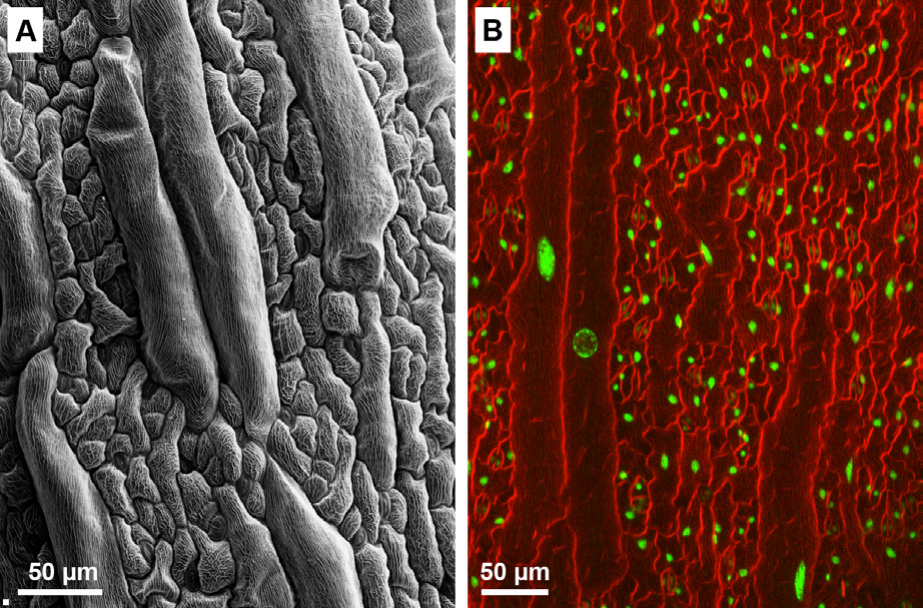
**The diversity of cell sizes in the *Arabidopsis *sepal epidermis**. **(a) **A scanning electron micrograph (SEM) of the sepal epidermis shows large giant cells are interspersed between smaller cells in a range of sizes. **(b) **A confocal maximum intensity projection of the sepal epidermis in which the cells are outlined in red by the plasma membrane dye FM4-64 revealing the variation in cell sizes. The nuclear size shown in green (*ML1::H2B-mYFP*) corresponds with the ploidy. The correlation between cell size and ploidy is evident: large cells have large nuclei indicating that they have undergone endoreduplciation whereas small cells have small nuclei indicating that they have remained diploid. Scale bars represent 50 μm.

All of these cell size control pathways can be developmentally regulated with temporal, tissue and spatial specificity to produce a variety of different cell sizes, often corresponding to specialized cellular functions. Cell size is regulated by transcription factors and co-activators such as GROWTH-REGULATING FACTOR (GRF) [198,199] and ANGUSTIFOLIA3 (AN3) [200,201]. Furthermore, cell size is actively patterned in different plant organs. For example, the *Arabidopsis *sepal epidermis has a broad diversity of cell sizes, from small cells with one-hundredth the length of the sepal to giant cells with one-fifth the length of the sepal (Figure 8a,b). This pattern forms as a result of the stochastic entry of cells into endoreduplication at different times [162]. The pattern is also regulated by the epidermal specification pathway, which promotes the formation of giant cells [116]. Giant cells are found on the back (abaxial) epidermis of *Arabidopsis *sepals and leaves, whereas the epidermal cells in the petal blade do not endoreduplicate and consequently they have a uniform small size.

**Figure 9:**
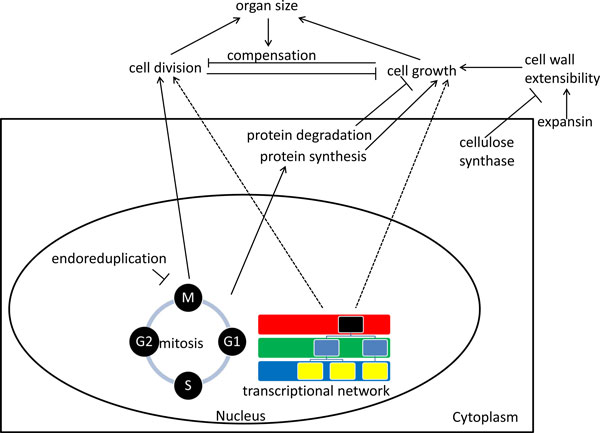
**Overview of cell size control in plants**. Plant cell size is determined by the growth of the cell versus its division. Cell growth is determined by macromolecular synthesis as well as expansion of the cell wall. Future challenges include untangling the regulatory network between the various pathways regulating the cell cycle, endoreduplication, the cell wall, and synthesis of cytoplasm, to elucidate how this crosstalk determines the ultimate cell size.

In summary, cell size control in plants is a highly dynamic and complicated process involving multiple biological pathways (Figure 9). Although many individual cell size regulators in each of these pathways have been discovered, a future challenge will be determining how these pathways integrate to form a complete cell size control network. Two recent advances are likely to guide the construction of such a network [202]. First, live imaging of cells in developing organs will allow us to determine the exact timing and detailed mechanism through which each player affects cell division, cell growth, and cell expansion. For example, the transcription factor JAGGED (JAG) regulates proliferation to control lateral organ shape and size in *Arabidopsis *sepals and petals [203,204]. Recently, by using three-dimensional live imaging, Schiessl *et al*. [205] discovered that *JAG *regulates the transition between tight coordination of cell volume with initiation of S phase of the cell cycle in the stem cells of the meristem and loose coordination in the initiating organ primordium. Ectopic expression of *JAG *in the floral meristem stem cells bypasses this tight cell size checkpoint, resulting in smaller cells entering S phase [205]. Second, computational modeling will allow us to predict the cumulative effect of multiple pathways acting simultaneously and feeding back on one another [206]. For example, while a diagram can be used to conceptualize the increase in cell size caused by one cell entering endoreduplication earlier than its neighbors [207], a computational model can expand this analysis to about 1,400 cells entering endoreduplication or dividing at stochastic times to pattern the entire sepal [162]. Such an integrated understanding may finally show how plant cells increase in size to compensate for decreased cell numbers to achieve consistent organ size.

## References

[B1] Rappé MS Connon SA Vergin KL Giovannoni SJ Cultivation of the ubiquitous SAR11 marine bacterioplankton clade Nature 2002 418 630 633 10.1038/nature00917 12167859

[B2] Schulz HN Jorgensen BB Big bacteria Annu Rev Microbiol 2001 55 105 137 10.1146/annurev.micro.55.1.105 11544351

[B3] Bailey JV Salman V Rouse GW Schulz-Vogt HN Levin LA Orphan VJ Dimorphism in methane seep-dwelling ecotypes of the largest known bacteria Isme J 2011 5 1926 1935 10.1038/ismej.2011.66 21697959 PMC3223306

[B4] Mendell JE Clements KD Choat JH Angert ER Extreme polyploidy in a large bacterium Proc Nat Acad Sci USA 2008 105 6730 6734 10.1073/pnas.0707522105 18445653 PMC2373351

[B5] Young KD The selective value of bacterial shape Microbiol Mol Biol Rev 2006 70 660 703 10.1128/MMBR.00001-06 16959965 PMC1594593

[B6] National Research Council Space Studies Board Size limits of very small microorganisms: Proceedings of a Workshop 1999 Washington, DC: National Academic Press 25077232

[B7] Beveridge TJ The bacterial surface: general considerations towards design and function Can J Microbiol 1988 34 363 372 10.1139/m88-067 3052749

[B8] Wagner JK Setayeshgar S Sharon LA Reilly JP Brun YV A nutrient uptake role for bacterial cell envelope extensions Proc Natl Acad Sci USA 2006 103 11772 11777 10.1073/pnas.0602047103 16861302 PMC1544245

[B9] Kalanetra KM Joye SB Sunseri NR Nelson DC Novel vacuolate sulfur bacteria from the Gulf of Mexico reproduce by reductive division in three dimensions Environ Microbiol 2005 7 1451 1460 10.1111/j.1462-2920.2005.00832.x 16104867

[B10] Young KD Bacterial shape Mol Microbiol 2003 49 571 580 12914007 10.1046/j.1365-2958.2003.03607.x

[B11] Young KD Bacterial shape: two-dimensional questions and possibilities Annu Rev Microbiol 2010 64 223 240 10.1146/annurev.micro.112408.134102 20825347 PMC3559087

[B12] den Blaauwen T de Pedro MA Nguyen-Disteche M Ayala JA Morphogenesis of rod-shaped sacculi FEMS Microbiol Rev 2008 32 321 344 10.1111/j.1574-6976.2007.00090.x 18291013

[B13] Margolin W Sculpting the bacterial cell Curr Biol 2009 19 R812 R822 10.1016/j.cub.2009.06.033 19906583 PMC4080913

[B14] Ausmees N Kuhn JR Jacobs-Wagner C The bacterial cytoskeleton: an intermediate filament-like function in cell shape Cell 2003 115 705 713 10.1016/S0092-8674(03)00935-8 14675535

[B15] Bagchi S Tomenius H Belova LM Ausmees N Intermediate filament-like proteins in bacteria and a cytoskeletal function in *Streptomyces* Mol Microbiol 2008 70 1037 1050 18976278 10.1111/j.1365-2958.2008.06473.x PMC2680258

[B16] Romberg L Levin PA Assembly dynamics of the bacterial cell division protein FtsZ: poised at the edge of stability Annu Rev Microbiol 2003 57 125 154 10.1146/annurev.micro.57.012903.074300 14527275 PMC5517307

[B17] Addinall SG Holland B The tubulin ancestor, FtsZ, draughtsman, designer and driving force for bacterial cytokinesis J Mol Biol 2002 318 219 236 10.1016/S0022-2836(02)00024-4 12051832

[B18] Graumann PL Cytoskeletal elements in bacteria Annu Rev Microbiol 2007 61 589 618 10.1146/annurev.micro.61.080706.093236 17506674

[B19] Lowe J van den Ent F Amos LA Molecules of the bacterial cytoskeleton Annu Rev Biophys Biomol Struct 2004 33 177 198 10.1146/annurev.biophys.33.110502.132647 15139810

[B20] Ingerson-Mahar M Gitai Z A growing family: the expanding universe of the bacterial cytoskeleton FEMS Microbiol Rev 2012 36 256 266 10.1111/j.1574-6976.2011.00316.x 22092065 PMC4114309

[B21] Barry RM Gitai Z Self-assembling enzymes and the origins of the cytoskeleton Curr Opin Microbiol 2011 14 704 711 10.1016/j.mib.2011.09.015 22014508 PMC3234109

[B22] Rothfield L Taghbalout A Shih YL Spatial control of bacterial division-site placement Nat Rev Microbiol 2005 3 959 968 10.1038/nrmicro1290 16322744

[B23] Lutkenhaus J Min oscillation in bacteria Adv Exp Med Biol 2008 641 49 61 18783171 10.1007/978-0-387-09794-7_4

[B24] Wu LJ Errington J Nucleoid occlusion and bacterial cell division Nat Rev Microbiol 2012 10 8 12 22020262 10.1038/nrmicro2671

[B25] Chien AC Hill NS Levin PA Cell size control in bacteria Curr Biol 2012 22 R340 349 10.1016/j.cub.2012.02.032 22575476 PMC3350639

[B26] Weart RB Lee AH Chien AC Haeusser DP Hill NS Levin PA A metabolic sensor governing cell size in bacteria Cell 2007 130 335 347 10.1016/j.cell.2007.05.043 17662947 PMC1971218

[B27] van Teeffelen S Wang S Furchtgott L Huang KC Wingreen NS Shaevitz JW Gitai Z The bacterial actin MreB rotates, and rotation depends on cell-wall assembly Proc Natl Acad Sci USA 2011 108 15822 15827 10.1073/pnas.1108999108 21903929 PMC3179079

[B28] Garner EC Bernard R Wang W Zhuang X Rudner DZ Mitchison T Coupled, circumferential motions of the cell wall synthesis machinery and MreB filaments in *B. subtilis* Science 2011 333 222 225 10.1126/science.1203285 21636745 PMC3235694

[B29] Dominguez-Escobar J Chastanet A Crevenna AH Fromion V Wedlich-Soldner R Carballido-Lopez R Processive movement of MreB-associated cell wall biosynthetic complexes in bacteria Science 2011 333 225 228 10.1126/science.1203466 21636744

[B30] Conlon I Raff M Differences in the way a mammalian cell and yeast cells coordinate cell growth and cell-cycle progression J Biol 2003 2 7 10.1186/1475-4924-2-7 12733998 PMC156598

[B31] Turner JJ Ewald JC Skotheim JM Cell size control in yeast Curr Biol 2012 22 R350 359 10.1016/j.cub.2012.02.041 22575477 PMC3350643

[B32] Mitchison Creanor Induction synchrony in the fission yeast Schizosaccharomyces pombe Exp Cell Res 1971 67 368 374 10.1016/0014-4827(71)90421-6 4255493

[B33] Johnston, Pringle, Hartwell Coordination of growth with cell division in the yeast saccharomyces cerevisiae Exp Cell Res 1977 105 79 98 10.1016/0014-4827(77)90154-9 320023

[B34] Elliott S McLaughlin C Rate of macromolecular synthesis through the cell cycle of the yeast Saccharomyces cerevisiae Proc Natl Acad Sci USA 1978 75 4384 4388 10.1073/pnas.75.9.4384 360219 PMC336119

[B35] Godin M Delgado FF Son S Grover WH Bryan AK Tzur A Jorgensen P Payer K Grossman AD Kirschner MW Manalis SR Using buoyant mass to measure the growth of single cells Nat Methods 2010 7 387 390 10.1038/nmeth.1452 20383132 PMC2862099

[B36] Creanor J Mitchison JM Patterns of protein synthesis during the cell cycle of the fission yeast Schizosaccharomyces pombe J Cell Sci 1982 58 263 285 7183688 10.1242/jcs.58.1.263

[B37] Nurse P Thuraux P Nasmyth K Genetic control of the cell division cycle in the fission yeast Schizosaccharomyces pombe Molec Gen Genet 1976 146 167 178 10.1007/BF00268085 958201

[B38] Fantes P Nurse P Control of cell size at division in fission yeast by a growth-modulated size control over nuclear division Exp Cell Res 1977 107 377 386 10.1016/0014-4827(77)90359-7 872891

[B39] Nurse P Genetic control of cell size at cell division in yeast Nature 1975 256 547 551 10.1038/256547a0 1165770

[B40] Nurse P Thuraux P Controls over the timing of DNA replication during the cell cycle of fission yeast Exp Cell Res 1977 107 365 375 10.1016/0014-4827(77)90358-5 872890

[B41] Hartwell LH Saccharomyces cerevisiae cell cycle Bacteriol Rev 1974 38 164 198 4599449 10.1128/br.38.2.164-198.1974 PMC413849

[B42] Nurse P Universal control mechanism regulating onset of M-phase Nature 1990 344 503 508 10.1038/344503a0 2138713

[B43] Russell P Nurse P cdc25+ functions as an inducer in the mitotic control of fission yeast Cell 1986 45 145 153 10.1016/0092-8674(86)90546-5 3955656

[B44] Russell P Nurse P Negative regulation of mitosis by wee1+, a gene encoding a protein kinase homolog Cell 1987 49 559 567 10.1016/0092-8674(87)90458-2 3032459

[B45] Gould L Nurse P Tyrosine phosphorylation of the fission yeast cdc2+ protein kinase regulates entry into mitosis Nature 1989 342 39 45 10.1038/342039a0 2682257

[B46] Lundgren K Walworth N Booher R Dembski M Kirschner M Beach D mik1 and wee1 cooperate in the inhibitory tyrosine phosphorylation of cdc2 Cell 1991 64 1111 1122 10.1016/0092-8674(91)90266-2 1706223

[B47] Moseley JB Mayeux A Paoletti A Nurse P A spatial gradient coordinates cell size and mitotic entry in fission yeast Nature 2009 459 857 860 10.1038/nature08074 19474789

[B48] Martin SG Berthelot-Grosjean M Polar gradients of the DYRK-family kinase Pom1 couple cell length with the cell cycle Nature 2009 459 852 856 10.1038/nature08054 19474792

[B49] Hachet O Berthelot-Grosjean M Kokkoris K Vincenzetti V Moosbrugger J Martin SG A phosphorylation cycle shapes gradients of the DYRK family kinase Pom1 at the plasma membrane Cell 2011 145 1116 1128 10.1016/j.cell.2011.05.014 21703453

[B50] Coudreuse D Nurse P Driving the cell cycle with a minimal CDK control network Nature 2010 468 1074 1079 10.1038/nature09543 21179163

[B51] Futcher B Cyclins and the wiring of the yeast cell cycle Yeast 1996 12 1635 1646 10.1002/(SICI)1097-0061(199612)12:16<1635::AID-YEA83>3.0.CO;2-O 9123966

[B52] Rupes I Checking cell size in yeast Trends Genet 2002 18 479 485 10.1016/S0168-9525(02)02745-2 12175809

[B53] Nash R Tokiwa G Sukhuijt A Erickson K Futcher B The WHI1+ gene of Saccharomyces cerevisiae tethers cell division to cell size and is a cyclin homolog EMBO J 1988 7 4335 4346 2907481 10.1002/j.1460-2075.1988.tb03332.x PMC455150

[B54] Cross FR DAF1, a mutant gene affecting size control, pheromone arrest, and cell cycle kinetics of Saccharomyces cerevisiae Mol Cell Biol 1988 8 4675 4684 3062366 10.1128/mcb.8.11.4675-4684.1988 PMC365557

[B55] Tyers M Tokiwa G Futcher B Comparison of the Saccharomyces cerevisiae G1 cyclins: Cln3 may bean upstreamactivator of Cln1, Cln2 and other cyclins EMBO J 1993 12 1955 1968 8387915 10.1002/j.1460-2075.1993.tb05845.x PMC413417

[B56] Tyers M Tokiwal G Nash R Futcher B The Cln3 - Cdc28 kinase complex of S.cerevisiae is regulated by proteolysis and phosphorylation EMBO J 1992 11 1773 1784 1316273 10.1002/j.1460-2075.1992.tb05229.x PMC556635

[B57] Polymenis M Schmidt EV Coupling of cell division to cell growth by translational control of the G1 cyclin CLN3 in yeast Genes Dev 1997 11 2522 2531 10.1101/gad.11.19.2522 9334317 PMC316559

[B58] de Bruin RA McDonald WH Kalashnikova TI Yates J Wittenberg C Cln3 activates G1-specific transcription via phosphorylation of the SBF bound repressor Whi5 Cell 2004 117 887 898 10.1016/j.cell.2004.05.025 15210110

[B59] Costanzo M Nishikawa JL Tang X Millman JS Schub O Breitkreuz K Dewar D Rupes I Andrews B Tyers M CDK activity antagonizes Whi5, an inhibitor of G1/S transcription in yeast Cell 2004 117 899 913 15210111 10.1016/j.cell.2004.05.024

[B60] Neumann FR Nurse P Nuclear size control in fission yeast J Cell Biol 2007 179 593 600 10.1083/jcb.200708054 17998401 PMC2080919

[B61] Jorgensen P Edgington NP Schneider BL Rupes I Tyers M Futcher B The size of the nucleus increases as yeast cells grow Mol Biol Cell 2007 18 3523 3532 10.1091/mbc.E06-10-0973 17596521 PMC1951755

[B62] Nurse P Cavalier-Smith T The Genetic Control of Cell Volume The Evolution of Genome Size 1985 Chichester: Wiley 185 196.63

[B63] Gregory TR Coincidence, coevolution, or causation? DNA content, cell size, and the C-value enigma Biol Rev Camb Philos Soc 2001 76 65 101 10.1017/S1464793100005595 11325054

[B64] Mortimer RK Radiobiological and genetic studies on a polyploid series (haploid to hexaploid) of Saccharomyces cerevisiae Radiation Res 1958 9 312 326 10.2307/3570795 13579200

[B65] Fantes PA Grant WD Pritchard RH Sudbery PE Wheals AE The regulation of cell size and the control of mitosis J Theor Biol 1975 50 213 244 10.1016/0022-5193(75)90034-X 1127959

[B66] Donachie WD Relationship between cell size and time of initiation of DNA replication Nature 1968 219 1077 1079 10.1038/2191077a0 4876941

[B67] Wang H Carey LB Cai Y Wijnen H Futcher B Recruitment of Cln3 cyclin to promoters controls cell cycle entry via histone deacetylase and other targets PLoS Biol 2009 7 e1000189 10.1371/journal.pbio.1000189 19823669 PMC2730028

[B68] Hara Y Kimura A Cell-size-dependent control of organelle sizes during development Results Probl Cell Differ 2011 53 93 108 10.1007/978-3-642-19065-0_5 21630142

[B69] Hara Y Kimura A Cell-size-dependent spindle elongation in the Caenorhabditis elegans early embryo Curr Biol 2009 19 1549 1554 10.1016/j.cub.2009.07.050 19682904

[B70] Carvalho A Desai A Oegema K Structural memory in the contractile ring makes the duration of cytokinesis independent of cell size Cell 2009 137 926 937 10.1016/j.cell.2009.03.021 19490897

[B71] Greenan G Brangwynne CP Jaensch S Gharakhani J Julicher F Hyman AA Centrosome size sets mitotic spindle length in Caenorhabditis elegans embryos Curr Biol 2010 20 353 358 10.1016/j.cub.2009.12.050 20137951

[B72] Siller KH Doe CQ Spindle orientation during asymmetric cell division Nat Cell Biol 2009 11 365 374 10.1038/ncb0409-365 19337318

[B73] Kellogg DR Moritz M Alberts BM The centrosome and cellular organization Annu Rev Biochem 1994 63 639 674 10.1146/annurev.bi.63.070194.003231 7979251

[B74] Grill SW Hyman AA Spindle positioning by cortical pulling forces Dev Cell 2005 8 461 465 10.1016/j.devcel.2005.03.014 15809029

[B75] Minc N Burgess D Chang F Influence of cell geometry on division-plane positioning Cell 2011 144 414 426 10.1016/j.cell.2011.01.016 21295701 PMC3048034

[B76] Strome S Wood WB Generation of asymmetry and segregation of germ-line granules in early C. elegans embryos Cell 1983 35 15 25 10.1016/0092-8674(83)90203-9 6684994

[B77] Reinsch S Gönczy P Mechanisms of nuclear positioning J Cell Sci 1998 111 2283 2295 9683624 10.1242/jcs.111.16.2283

[B78] Hamaguchi MS Hiramoto Y Analysis of the role of astral rays in pronuclear migration in sand dollar eggs by the colcemid-UV method Dev Growth Differ 1986 28 143 156 10.1111/j.1440-169X.1986.00143.x 37280905

[B79] Holy TE Dogterom M Yurke B Leibler S Assembly and positioning of microtubule asters in microfabricated chambers Proc Natl Acad Sci USA 1997 94 6228 6231 10.1073/pnas.94.12.6228 9177199 PMC21031

[B80] Tran PT Marsh L Doye V Inoue S Chang F A mechanism for nuclear positioning in fission yeast based on microtubule pushing J Cell Biol 2001 153 397 411 10.1083/jcb.153.2.397 11309419 PMC2169469

[B81] Kimura A Onami S Computer simulations and image processing reveal length-dependent pulling force as the primary mechanism for C. elegans male pronuclear migration Dev Cell 2005 8 765 775 10.1016/j.devcel.2005.03.007 15866166

[B82] Vallee RB Stehman SA How dynein helps the cell find its center: a servomechanical model Trends Cell Biol 2005 15 288 294 10.1016/j.tcb.2005.04.005 15953546

[B83] Dogterom M Kerssemakers JW Romet-Lemonne G Janson ME Force generation by dynamic microtubules Curr Opin Cell Biol 2005 17 67 74 10.1016/j.ceb.2004.12.011 15661521

[B84] Kimura A Onami S Modeling microtubule-mediated forces and centrosome positioning in Caenorhabditis elegans embryos Methods Cell Biol 2010 97 437 453 20719284 10.1016/S0091-679X(10)97023-4

[B85] Zhu J Burakov A Rodionov V Mogilner A Finding the cell center by a balance of dynein and myosin pulling and microtubule pushing: a computational study Mol Biol Cell 2010 21 4418 4427 10.1091/mbc.E10-07-0627 20980619 PMC3002394

[B86] Kimura K Kimura A Intracellular organelles mediate cytoplasmic pulling force for centrosome centration in the Caenorhabditis elegans early embryo Proc Natl Acad Sci USA 2011 108 137 142 10.1073/pnas.1013275108 21173218 PMC3017145

[B87] Kimura K Kimura A A novel mechanism of microtubule length-dependent force to pull centrosomes toward the cell center BioArchitecture 2011 1 74 79 10.4161/bioa.1.2.15549 21866267 PMC3158624

[B88] Laan L Pavin N Husson J Romet-Lemonne G van Duijn M Lopez MP Vale RD Julicher F Reck-Peterson SL Dogterom M Cortical dynein controls microtubule dynamics to generate pulling forces that position microtubule asters Cell 2012 148 502 514 10.1016/j.cell.2012.01.007 22304918 PMC3292199

[B89] Wühr M Tan ES Parker SK Detrich HW Mitchison TJ A model for cleavage plane determination in early amphibian and fish embryos Curr Biol 2010 20 2040 2045 10.1016/j.cub.2010.10.024 21055946 PMC3031131

[B90] Mitchison T Kirschner M Dynamic instability of microtubule growth Nature 1984 312 237 242 10.1038/312237a0 6504138

[B91] Howard J Mechanics of Motor Proteins and the Cytoskeleton 2001 Massachusetts: Sinauer Associates

[B92] Walker RA O'Brien ET Pryer NK Soboeiro MF Voter WA Erickson HP Salmon ED Dynamic instability of individual microtubules analyzed by video light microscopy: rate constants and transition frequencies J Cell Biol 1988 107 1437 1448 10.1083/jcb.107.4.1437 3170635 PMC2115242

[B93] Cassimeris L Pryer NK Salmon ED Real-time observations of microtubule dynamic instability in living cells J Cell Biol 1988 107 2223 2231 10.1083/jcb.107.6.2223 3198684 PMC2115680

[B94] Soltys BJ Borisy GG Polymerization of tubulin in vivo: direct evidence for assembly onto microtubule ends and from centrosomes J Cell Biol 1985 100 1682 1689 10.1083/jcb.100.5.1682 3886672 PMC2113852

[B95] Albertson DG Formation of the first cleavage spindle in nematode embryos Dev Biol 1984 101 61 72 10.1016/0012-1606(84)90117-9 6692980

[B96] Gönczy P Bellanger JM Kirkham M Pozniakowski A Baumer K Phillips JB Hyman AA zyg-8, a gene required for spindle positioning in C. elegans, encodes a doublecortin-related kinase that promotes microtubule assembly Dev Cell 2001 1 363 375 10.1016/S1534-5807(01)00046-6 11702948

[B97] Wühr M Dumont S Groen AC Needleman DJ Mitchison TJ How does a millimeter-sized cell find its center? Cell Cycle 2009 8 1115 1121 10.4161/cc.8.8.8150 19282671 PMC2880816

[B98] Dogterom M Yurke B Microtubule dynamics and the positioning of microtubule organizing centers Phys Rev Lett 1998 81 485 488 10.1103/PhysRevLett.81.485

[B99] Faivre-Moskalenko C Dogterom M Dynamics of microtubule asters in microfabricated chambers: the role of catastrophes Proc Natl Acad Sci USA 2002 99 16788 16793 10.1073/pnas.252407099 12486218 PMC139222

[B100] Neurohr G Naegeli A Titos I Theler D Greber B Diez J Gabaldon T Mendoza M Barral Y A midzone-based ruler adjusts chromosome compaction to anaphase spindle length Science 2011 332 465 468 10.1126/science.1201578 21393511

[B101] Ladouceur AM Ranjan R Maddox PS Cell size: chromosomes get slapped by a midzone ruler Curr Biol 2011 21 R388 390 10.1016/j.cub.2011.04.009 21601795

[B102] Grill SW Gönczy P Stelzer EH Hyman AA Polarity controls forces governing asymmetric spindle positioning in the Caenorhabditis elegans embryo Nature 2001 409 630 633 10.1038/35054572 11214323

[B103] Mogilner A Wollman R Civelekoglu-Scholey G Scholey J Modeling mitosis Trends Cell Biol 2006 16 88 96 10.1016/j.tcb.2005.12.007 16406522

[B104] Wilson EB The Cell in Development and Heredity 1934 New York: MacMillan

[B105] Hämmerling J Nucleo-cytoplasmic interactions in Acetabularia and other cells Annu Rev Plant Physiol 1963 14 65 92 10.1146/annurev.pp.14.060163.000433

[B106] Matz MV Frank TM Marshall NJ Widder EA Johnsen S Giant deep-sea protist produces bilaterian-like traces Curr Biol 2008 18 1849 1854 10.1016/j.cub.2008.10.028 19026540

[B107] Jacobs WP Caulerpa Sci Am 1994 271 100 105

[B108] Arnold ZM Observations on the sexual generation of Gromia oviformis Dujardin J Protozool 1966 13 23 27 5912388 10.1111/j.1550-7408.1966.tb01863.x

[B109] Crawley J Some observations on the fine structure of the gametes and zygotes of Acetabularia Planta 1966 69 365 376 10.1007/BF00392287 24557887

[B110] Goldstein M Morrall S Gametogenesis and fertilization in Caulerpa Ann N Y Acad Sci 1970 175 660 672 10.1111/j.1749-6632.1970.tb45183.x

[B111] Smirnov A Nassonova E Berney C Fahrni J Bolivar I Pawlowski J Molecular phylogeny and classification of the lobose amoebae Protist 2005 156 129 142 10.1016/j.protis.2005.06.002 16171181

[B112] Rogerson A Patterson DJ The naked ramicristate amoebae (Gymnamoebae) An Illustrated Guide to the Protozoa 2002 2 Lawrence, Kansas: Society of Protozoologists 1023 1053

[B113] Chalkley H The observation of mitosis in the living cell in Amoeba proteus Science 1934 80 208 209 10.1126/science.80.2070.208 17747194

[B114] Kudo R Pelomyxa carolinensis Wilson. II. Nuclear division and plasmotomy J Morphol 1947 80 93 143 10.1002/jmor.1050800105 20281548

[B115] Kudo R Pelomyxa carolinensis Wilson. III. Further observations on plasmotomy J Morphol 1949 85 163 176 10.1002/jmor.1050850107 18135698

[B116] Roeder AH Cunha A Ohno CK Meyerowitz EM Cell cycle regulates cell type in the Arabidopsis sepal Development 2012 139 4416 4427 10.1242/dev.082925 23095885

[B117] Morgan TH Regeneration of proportionate structures in Stentor Biol Bull 1901 2 311 328 10.2307/1535709

[B118] Tartar V The Biology of Stentor 1961 Oxford: Pergamon Press

[B119] Tartar V Reactions of Stentor coeruleus to homoplastic grafting J Exp Zool 1954 127 511 575 10.1002/jez.1401270306

[B120] Tartar V Reconstitution of minced Stentor coeruleus J Exp Zool 1960 144 187 207 10.1002/jez.1401440208 13775373

[B121] Uhlig G Entwicklungsphysiologische Untersuchungen zur Morphogenese von Stentor coeruleus Ehrbg Arch Protistentenk 1960 105 1 109

[B122] Tartar V Morphogenesis in homopolar tandem grafted Stentor coeruleus J Exp Zool 1964 156 243 251 10.1002/jez.1401560302 14197064

[B123] Elinson RP Fertilization and aqueous development of the puerto rican terrestrial-breeding frog, Eleutherodactylus coqui J Morphol 1987 193 217 224 10.1002/jmor.1051930208 29907000

[B124] Collazo A Keller R Early development of Ensatina eschscholtzii: an amphibian with a large, yolky egg EvoDevo 2010 1 6 10.1186/2041-9139-1-6 20849648 PMC2938725

[B125] Elinson RP del Pino EM Developmental diversity of amphibians Wiley Interdiscip Rev Membr Transp Signal 2012 1 345 369 22662314 10.1002/wdev.23 PMC3364608

[B126] Summers K Sea McKeon C Heying H The evolution of parental care and egg size: a comparative analysis in frogs Proc Biol Sci 2006 273 687 692 10.1098/rspb.2005.3368 16608687 PMC1560067

[B127] del Pino EM Loor-Vela S The pattern of early cleavage of the marsupial frog Gastrotheca riobambae Development 1990 110 781 789 2088720 10.1242/dev.110.3.781

[B128] Gould SJ A developmental constraint in Cerion, with comments of the definition and interpretation of constraint in evolution Evolution 1989 43 516 539 10.2307/2409056 28568388

[B129] Jenkins R Fine structure of division in ciliate protozoa I. Micronuclear mitosis in Blepharisma J Cell Biol 1967 34 463 481 10.1083/jcb.34.2.463 4962327 PMC2107323

[B130] Tartar V Micrurgical experiments on cytokinesis in Stentor coeruleus J Exp Zool 1968 167 21 35 10.1002/jez.1401670103 4967202

[B131] Gilbert S Developmental Biology 2006 8 Sunderland, MA: Sinauer Associates

[B132] Su TT O'Farrell PH Size control: cell proliferation does not equal growth Curr Biol 1998 8 R687 689 10.1016/S0960-9822(98)70436-1 9768354 PMC2754256

[B133] Kozma SC Thomas G Regulation of cell size in growth, development and human disease: PI3K, PKB and S6K Bioessays 2002 24 65 71 10.1002/bies.10031 11782951

[B134] Fankhauser G Maintenance of normal structure in heteroploid salamander larvae, through compensation of changes in cell size by adjustment of cell number and cell shape J Exp Zool 1945 100 445 455 10.1002/jez.1401000310 21010861

[B135] Nieuwkoop PD Faber J Normal Table of Xenopus laevis (Daudin) 1994 3 New York: Garland

[B136] Danilchik MV Bedrick SD Brown EE Ray K Furrow microtubules and localized exocytosis in cleaving Xenopus laevis embryos J Cell Sci 2003 116 273 283 10.1242/jcs.00217 12482913

[B137] Wuhr M Chen Y Dumont S Groen AC Needleman DJ Salic A Mitchison TJ Evidence for an upper limit to mitotic spindle length Curr Biol 2008 18 1256 1261 10.1016/j.cub.2008.07.092 18718761 PMC2561182

[B138] Wang P Hayden S Masui Y Transition of the blastomere cell cycle from cell size-independent to size-dependent control at the midblastula stage in Xenopus laevis J Exp Zool 2000 287 128 144 10.1002/1097-010X(20000701)287:2<128::AID-JEZ3>3.0.CO;2-G 10900432

[B139] Newport J Kirschner M A major developmental transition in early Xenopus embryos: II. Control of the onset of transcription Cell 1982 30 687 696 10.1016/0092-8674(82)90273-2 7139712

[B140] Newport J Kirschner M A major developmental transition in early Xenopus embryos: I. characterization and timing of cellular changes at the midblastula stage Cell 1982 30 675 686 10.1016/0092-8674(82)90272-0 6183003

[B141] Levy DL Heald R Nuclear size is regulated by importin alpha and Ntf2 in Xenopus Cell 2010 143 288 298 10.1016/j.cell.2010.09.012 20946986 PMC2966892

[B142] Brown KS Blower MD Maresca TJ Grammer TC Harland RM Heald R Xenopus tropicalis egg extracts provide insight into scaling of the mitotic spindle J Cell Biol 2007 176 765 770 10.1083/jcb.200610043 17339377 PMC2064050

[B143] Woolner S O'Brien LL Wiese C Bement WM Myosin-10 and actin filaments are essential for mitotic spindle function J Cell Biol 2008 182 77 88 10.1083/jcb.200804062 18606852 PMC2447898

[B144] Loughlin R Wilbur JD McNally FJ Nedelec FJ Heald R Katanin contributes to interspecies spindle length scaling in Xenopus Cell 2011 147 1397 1407 10.1016/j.cell.2011.11.014 22153081 PMC3240848

[B145] Lecuit T Lenne PF Cell surface mechanics and the control of cell shape, tissue patterns and morphogenesis Nat Rev Mol Cell Biol 2007 8 633 644 17643125 10.1038/nrm2222

[B146] Keller R Developmental biology. Physical biology returns to morphogenesis Science 2012 338 201 203 10.1126/science.1230718 23066066

[B147] Fine LG Norman J Cellular events in renal hypertrophy Annu Rev Physiol 1989 51 19 32 10.1146/annurev.ph.51.030189.000315 2469382

[B148] Lienkamp S Liu K Karner C Carroll T Ronnenberger O Wallingford JB Walz G Kidney tubules elongate using a novel mode of planar cell polarity-dependent convergent extension Nat Genet 2012 in press 10.1038/ng.2452 PMC4167614 23143599

[B149] Karner CM Chirumamilla R Aoki S Igarashi P Wallingford JB Carroll TJ Wnt9b signaling regulates planar cell polarity and kidney tubule morphogenesis Nat Genet 2009 41 793 799 10.1038/ng.400 19543268 PMC2761080

[B150] Happe H van der Wal AM Leonhard WN Kunnen SJ Breuning MH de Heer E Peters DJ Altered Hippo signalling in polycystic kidney disease J Pathol 2011 224 133 142 10.1002/path.2856 21381034

[B151] Makita R Uchijima Y Nishiyama K Amano T Chen Q Takeuchi T Mitani A Nagase T Yatomi Y Aburatani H Nakagawa O Small EV Cobo-Stark P Igarashi P Murakami M Tominaga J Sato T Asano T Kurihara Y Kurihara H Multiple renal cysts, urinary concentration defects, and pulmonary emphysematous changes in mice lacking TAZ Am J Physiol Renal Physiol 2008 294 F542 553 10.1152/ajprenal.00201.2007 18172001

[B152] Chen JK Chen J Neilson EG Harris RC Role of mammalian target of rapamycin signaling in compensatory renal hypertrophy J Am Soc Nephrol 2005 16 1384 1391 10.1681/ASN.2004100894 15788477

[B153] Chen JK Chen J Thomas G Kozma SC Harris RC S6 kinase 1 knockout inhibits uninephrectomy- or diabetes-induced renal hypertrophy Am J Physiol Renal Physiol 2009 297 F585 593 10.1152/ajprenal.00186.2009 19474189 PMC2739710

[B154] Price H Sparrow A Nauman AF Correlations between nuclear volume, cell volume and DNA content in meristematic cells of herbaceous angiosperms Cell Mol Life Sci 1973 29 1028 1029 10.1007/BF01930444

[B155] Haber A Foard D Anatomical analysis of wheat growing without cell division Am J Bot 1961 4 438 446

[B156] Haber AH Carrier WL Foard DE Metabolic studies of gamma-irradiated wheat growing without cell division Am J Bot 1961 48 431 438 10.2307/2439444

[B157] Haber AH Foard DE Further studies of gamma-irradiated wheat and their relevance to use of mitotic inhibition for developmental studies Am J Bot 1964 51 151 159 10.2307/2440099

[B158] Foard DE Haber AH Fishman TN Initiation of lateral root primordia without completion of mitosis and without cytokinesis in uniseriate pericycle Am J Bot 1965 52 580 590 10.2307/2440119

[B159] Kelliher T Walbot V Emergence and patterning of the five cell types of the Zea mays anther locule Dev Biol 2011 350 32 49 10.1016/j.ydbio.2010.11.005 21070762 PMC3024885

[B160] Tsukaya H Organ shape and size: a lesson from studies of leaf morphogenesis Curr Opin Plant Biol 2003 6 57 62 10.1016/S1369526602000055 12495752

[B161] Asl LK Dhondt S Boudolf V Beemster GT Beeckman T Inze D Govaerts W De Veylder L Model-based analysis of Arabidopsis leaf epidermal cells reveals distinct division and expansion patterns for pavement and guard cells Plant Physiol 2011 156 2172 2183 10.1104/pp.111.181180 21693673 PMC3149966

[B162] Roeder AHK Chickarmane V Cunha A Obara B Manjunath BS Meyerowitz EM Variability in the control of cell division underlies sepal epidermal patterning in Arabidopsis thaliana PLoS Biol 2010 8 e1000367 10.1371/journal.pbio.1000367 20485493 PMC2867943

[B163] Dewitte W Riou-Khamlichi C Scofield S Healy JM Jacqmard A Kilby NJ Murray JA Altered cell cycle distribution, hyperplasia, and inhibited differentiation in Arabidopsis caused by the D-type cyclin CYCD3 Plant Cell 2003 15 79 92 10.1105/tpc.004838 12509523 PMC143452

[B164] Menges M Samland AK Planchais S Murray JA The D-type cyclin CYCD3;1 is limiting for the G1-to-S-phase transition in Arabidopsis Plant Cell 2006 18 893 906 10.1105/tpc.105.039636 16517759 PMC1425856

[B165] Hemerly A Engler Jde A Bergounioux C Van Montagu M Engler G Inze D Ferreira P Dominant negative mutants of the Cdc2 kinase uncouple cell division from iterative plant development EMBO J 1995 14 3925 3936 7664733 10.1002/j.1460-2075.1995.tb00064.x PMC394471

[B166] Bemis SM Torii KU Autonomy of cell proliferation and developmental programs during Arabidopsis aboveground organ morphogenesis Dev Biol 2007 304 367 381 10.1016/j.ydbio.2006.12.049 17258192

[B167] Eloy NB de Freitas Lima M Van Damme D Vanhaeren H Gonzalez N De Milde L Hemerly AS Beemster GT Inze D Ferreira PC The APC/C subunit 10 plays an essential role in cell proliferation during leaf development Plant J 2011 68 351 363 10.1111/j.1365-313X.2011.04691.x 21711400

[B168] Rojas CA Eloy NB Lima Mde F Rodrigues RL Franco LO Himanen K Beemster GT Hemerly AS Ferreira PC Overexpression of the Arabidopsis anaphase promoting complex subunit CDC27a increases growth rate and organ size Plant Mol Biol 2009 71 307 318 10.1007/s11103-009-9525-7 19629716

[B169] Wilson EB The Cell in Development and Heredity 1928 3 New York: Macmillan

[B170] Jorgensen P Tyers M How cells coordinate growth and division Curr Biol 2004 14 R1014 R1027 10.1016/j.cub.2004.11.027 15589139

[B171] Sugimoto-Shirasu K Roberts K "Big it up": endoreduplication and cell-size control in plants Curr Opin Plant Biol 2003 6 544 553 10.1016/j.pbi.2003.09.009 14611952

[B172] Melaragno JE Mehrotra B Coleman AW Relationship between endopolyploidy and cell size in epidermal tissue of Arabidopsis Plant Cell 1993 5 1661 1668 12271050 10.1105/tpc.5.11.1661 PMC160394

[B173] Qi R John PC Expression of genomic AtCYCD2;1 in Arabidopsis induces cell division at smaller cell sizes: implications for the control of plant growth Plant Physiol 2007 144 1587 1597 10.1104/pp.107.096834 17513485 PMC1914123

[B174] De Veylder L Beeckman T Beemster GT Krols L Terras F Landrieu I van der Schueren E Maes S Naudts M Inze D Functional analysis of cyclin-dependent kinase inhibitors of Arabidopsis Plant Cell 2001 13 1653 1668 11449057 10.1105/TPC.010087 PMC139548

[B175] Verkest A Manes CL Vercruysse S Maes S Van Der Schueren E Beeckman T Genschik P Kuiper M Inze D De Veylder L The cyclin-dependent kinase inhibitor KRP2 controls the onset of the endoreduplication cycle during Arabidopsis leaf development through inhibition of mitotic CDKA;1 kinase complexes Plant Cell 2005 17 1723 1736 10.1105/tpc.105.032383 15863515 PMC1143072

[B176] Churchman ML Brown ML Kato N Kirik V Hülskamp M Inzé D De Veylder L Walker JD Zheng Z Oppenheimer DG Gwin T Churchman J Larkin JC SIAMESE, a plant-specific cell cycle regulator, controls endoreplication onset in Arabidopsis thaliana Plant Cell 2006 18 3145 3157 10.1105/tpc.106.044834 17098811 PMC1693949

[B177] Walker JD Oppenheimer DG Concienne J Larkin JC SIAMESE, a gene controlling the endoreduplication cell cycle in Arabidopsis thaliana trichomes Development 2000 127 3931 3940 10952891 10.1242/dev.127.18.3931

[B178] Castellano MM del Pozo JC Ramirez-Parra E Brown S Gutierrez C Expression and stability of Arabidopsis CDC6 are associated with endoreplication Plant Cell 2001 13 2671 2686 11752380 10.1105/tpc.010329 PMC139481

[B179] Horvath BM Magyar Z Zhang Y Hamburger AW Bako L Visser RG Bachem CW Bogre L EBP1 regulates organ size through cell growth and proliferation in plants EMBO J 2006 25 4909 4920 10.1038/sj.emboj.7601362 17024182 PMC1618091

[B180] Kurepa J Smalle JA Structure, function and regulation of plant proteasomes Biochimie 2008 90 324 335 10.1016/j.biochi.2007.07.019 17825468

[B181] Kurepa J Wang S Li Y Zaitlin D Pierce AJ Smalle JA Loss of 26S proteasome function leads to increased cell size and decreased cell number in Arabidopsis shoot organs Plant Physiol 2009 150 178 189 10.1104/pp.109.135970 19321709 PMC2675745

[B182] Sonoda Y Sako K Maki Y Yamazaki N Yamamoto H Ikeda A Yamaguchi J Regulation of leaf organ size by the Arabidopsis RPT2a 19S proteasome subunit Plant J 2009 60 68 78 10.1111/j.1365-313X.2009.03932.x 19500299

[B183] Deprost D Yao L Sormani R Moreau M Leterreux G Nicolai M Bedu M Robaglia C Meyer C The Arabidopsis TOR kinase links plant growth, yield, stress resistance and mRNA translation EMBO Rep 2007 8 864 870 10.1038/sj.embor.7401043 17721444 PMC1973950

[B184] Hay N Sonenberg N Upstream and downstream of mTOR Genes Dev 2004 18 1926 1945 10.1101/gad.1212704 15314020

[B185] Wullschleger S Loewith R Hall MN TOR signaling in growth and metabolism Cell 2006 124 471 484 10.1016/j.cell.2006.01.016 16469695

[B186] Ren M Qiu S Venglat P Xiang D Feng L Selvaraj G Datla R Target of rapamycin regulates development and ribosomal RNA expression through kinase domain in Arabidopsis Plant Physiol 2011 155 1367 1382 10.1104/pp.110.169045 21266656 PMC3046592

[B187] Cosgrove DJ Growth of the plant cell wall Nat Rev Mol Cell Biol 2005 6 850 861 10.1038/nrm1746 16261190

[B188] Cosgrove DJ Wall extensibility: its nature, measurement and relationship to plant cell growth New Phytol 1993 124 1 23 10.1111/j.1469-8137.1993.tb03795.x 11537718

[B189] Cosgrove DJ Loosening of plant cell walls by expansins Nature 2000 407 321 326 10.1038/35030000 11014181

[B190] McQueen-Mason S Durachko DM Cosgrove DJ Two endogenous proteins that induce cell wall extension in plants Plant Cell 1992 4 1425 1433 11538167 10.1105/tpc.4.11.1425 PMC160229

[B191] Cho HT Cosgrove DJ Altered expression of expansin modulates leaf growth and pedicel abscission in Arabidopsis thaliana Proc Natl Acad Sci USA 2000 97 9783 9788 10.1073/pnas.160276997 10931949 PMC16942

[B192] Paredez AR Somerville CR Ehrhardt DW Visualization of cellulose synthase demonstrates functional association with microtubules Science 2006 312 1491 1495 10.1126/science.1126551 16627697

[B193] Ambrose JC Shoji T Kotzer AM Pighin JA Wasteneys GO The Arabidopsis CLASP gene encodes a microtubule-associated protein involved in cell expansion and division Plant Cell 2007 19 2763 2775 10.1105/tpc.107.053777 17873093 PMC2048705

[B194] Peremyslov VV Prokhnevsky AI Dolja VV Class XI myosins are required for development, cell expansion, and F-Actin organization in Arabidopsis Plant Cell 2010 22 1883 1897 10.1105/tpc.110.076315 20581304 PMC2910955

[B195] Bringmann M Li E Sampathkumar A Kocabek T Hauser MT Persson S POM-POM2/cellulose synthase interacting1 is essential for the functional association of cellulose synthase and microtubules in Arabidopsis Plant Cell 2012 24 163 177 10.1105/tpc.111.093575 22294619 PMC3289571

[B196] Elsner J Michalski M Kwiatkowska D Spatiotemporal variation of leaf epidermal cell growth: a quantitative analysis of Arabidopsis thaliana wild-type and triple cyclinD3 mutant plants Ann Bot 2012 109 897 910 10.1093/aob/mcs005 22307569 PMC3310487

[B197] Haber AH Nonessentiality of concurrent cell divisions for degree of polarization of leaf growth .1. Studies with radiation-induced mitotic inhibition Am J Bot 1962 49 583 589 10.2307/2439715

[B198] Kim JH Choi D Kende H The AtGRF family of putative transcription factors is involved in leaf and cotyledon growth in Arabidopsis Plant J 2003 36 94 104 10.1046/j.1365-313X.2003.01862.x 12974814

[B199] Lee BH Ko JH Lee S Lee Y Pak JH Kim JH The Arabidopsis GRF-INTERACTING FACTOR gene family performs an overlapping function in determining organ size as well as multiple developmental properties Plant Physiol 2009 151 655 668 10.1104/pp.109.141838 19648231 PMC2754652

[B200] Horiguchi G Kim GT Tsukaya H The transcription factor AtGRF5 and the transcription coactivator AN3 regulate cell proliferation in leaf primordia of Arabidopsis thaliana Plant J 2005 43 68 78 10.1111/j.1365-313X.2005.02429.x 15960617

[B201] Kim JH Kende H A transcriptional coactivator, AtGIF1, is involved in regulating leaf growth and morphology in Arabidopsis Proc Natl Acad Sci USA 2004 101 13374 13379 10.1073/pnas.0405450101 15326298 PMC516574

[B202] Roeder AH Tarr PT Tobin C Zhang X Chickarmane V Cunha A Meyerowitz EM Computational morphodynamics of plants: integrating development over space and time Nat Rev Mol Cell Biol 2011 12 265 273 10.1038/nrm3079 21364682 PMC4128830

[B203] Dinneny JR Yadegari R Fischer RL Yanofsky MF Weigel D The role of JAGGED in shaping lateral organs Development 2004 131 1101 1110 10.1242/dev.00949 14973282

[B204] Ohno CK Reddy GV Heisler MG Meyerowitz EM The Arabidopsis JAGGED gene encodes a zinc finger protein that promotes leaf tissue development Development 2004 131 1111 1122 10.1242/dev.00991 14973281

[B205] Schiessl K Kausika S Southam P Bush M Sablowski R JAGGED controls growth anisotropy and coordination between cell size and cell cycle during plant organogenesis Curr Biol 2012 22 1739 1746 10.1016/j.cub.2012.07.020 22902754 PMC3471073

[B206] Roeder AH When and where plant cells divide: a perspective from computational modeling Curr Opin Plant Biol 2012 15 638 644 10.1016/j.pbi.2012.08.002 22939706

[B207] Traas J Hulskamp M Gendreau E Hofte H Endoreduplication and development: rule without dividing? Curr Opin Plant Biol 1998 1 498 503 10.1016/S1369-5266(98)80042-3 10066638

